# Beyond borders: engineering organ-targeted immunotherapies to overcome site-specific barriers in cancer

**DOI:** 10.1007/s13346-025-01935-4

**Published:** 2025-08-11

**Authors:** Thrinayan Moorthy, Bhanu Nirosha Yalamandala, Thi My Hue Huynh, Hui-Wen Lien, Wan-Chi Pan, Hoi Man Iao, Yun-Hsuan Chang, Shang-Hsiu Hu

**Affiliations:** Department of Biomedical Engineering and Environmental Sciences, National Tsing Hua University, Hsinchu, 300044 Taiwan

**Keywords:** Lung cancer, Glioblastoma (GBM), Hepatocellular carcinoma (HCC), Nanovaccine, Immunotherapy, Cancer therapy

## Abstract

Organ-targeted immunotherapy is emerging as a transformative strategy to significantly improve the precision, efficacy, and safety of cancer immunotherapy. Tumors frequently arise in, or metastasize to, immunologically unique organs such as the brain, liver, and lungs—each presenting formidable barriers that impede uniform treatment success. These organs not only represent common metastatic sites but also host distinct immune microenvironments that demand customized therapeutic approaches. Nanovaccine-based immunotherapies have recently gained traction as a promising solution to overcome these organ-specific challenges. The brain, protected by the blood–brain barrier (BBB), limits immune cell infiltration and drug penetration, especially in aggressive cancers like glioblastoma (GBM). The liver, characterized by its immune-tolerant landscape and dense population of phagocytic cells, suppresses antitumor responses in hepatocellular carcinoma (HCC). Meanwhile, the lungs’ vast vasculature and continuous exposure to airborne antigens necessitate precision delivery strategies to trigger robust immunity without provoking excessive inflammation. To surmount these hurdles, nanocarriers have been engineered to leverage passive targeting through the enhanced permeability and retention (EPR) effect, active targeting via organ-specific ligands, immune cell hitchhiking, and stimuli-responsive release mechanisms. These innovations enable nanovaccines to elicit localized immune activation, reshape the tumor microenvironment (TME), and enhance cytotoxic T-cell responses. This review underscores the critical importance and urgent need for continued development of organ-specific nanovaccine platforms. It calls for intensified research efforts to translate these technologies into clinically viable therapies capable of addressing the most immunologically challenging tumor sites.

**Graphical Abstract:**

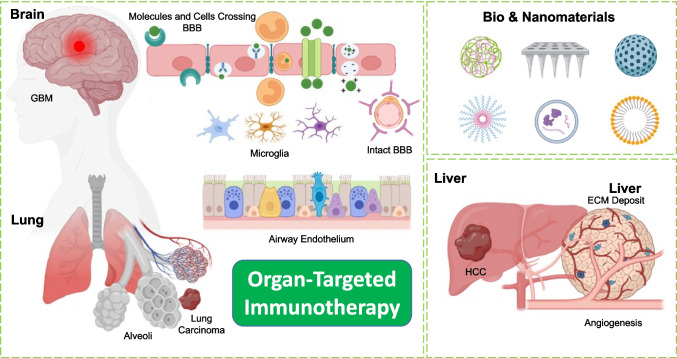

## Introduction

The quest to eradicate cancer has persisted for over a century with limited treatment success, prompting researchers to explore interventions offering a permanent cure. While conventional approaches involving surgical resection, chemotherapy, and radiotherapy remain standard-of-care and can offer significant benefits, especially when tumors are detected at early stages, their effectiveness is often limited in advanced or metastatic cancers due to therapy resistance and tumor immune evasion. This limited progress stems from malignant tumors adapting to evade therapy, involving metabolic rewiring that promotes unchecked proliferation and survival [1–3]. Moreover, the TME comprises complex immune cell networks, which tumors manipulate to avoid immune attack [4]. In response, researchers aim to harness the innate capabilities of immune cells to eliminate cancer. As a result, cancer immunotherapy has gained significant prominence by harnessing the immune system's power to combat tumors [5].

To address the limitations of systemic treatments, organ-targeted immunotherapy has emerged as a promising approach [6]. Given that each organ possesses unique structural and immunological features, generalized therapies often fall short [7]. Each organ presents unique physiological and pathological features that significantly impact targeted delivery strategies. The brain is shielded by the highly selective blood–brain barrier (BBB), a tightly regulated endothelial interface that limits the passage of most therapeutic agents and immune cells, posing a major obstacle for effective drug delivery and immunotherapy [8]. Meanwhile, the lung’s extensive alveolar-capillary network provides a large surface area for gas exchange but also creates a highly reactive immune environment, constantly balancing immune tolerance and activation in response to inhaled antigens and pathogens, complicating immune-targeted therapies [9]. The liver’s sinusoidal vasculature is characterized by fenestrated endothelial cells that facilitate high molecular exchange, while also contributing to its role as an immunotolerant organ, which promotes immune suppression that tumors can exploit [10]. Understanding these distinct barriers is critical for designing organ-specific immunotherapies that can effectively overcome local immunosuppression and improve treatment outcomes. Precision delivery of therapeutic agents enhances local immune activation while reducing systemic side effects [11]. This is particularly critical in immune-privileged organs like the brain and liver, where systemic immune activation is insufficient [12]. Targeting resident immune populations can reprogram suppressive environments and reinvigorate exhausted responses. Tailoring treatments to organ biology improves efficacy and marks a step toward personalized immunotherapy [13]. Aligned with these targeted strategies, the conception of immunotherapy can be categorized into two approaches: triggering and manipulation of immune cells/systems, and immune checkpoint-targeted therapy [14]. Both have delivered promising outcomes, enabling immune cells to identify and destroy tumors [15, 16]. Immune checkpoint inhibitors (ICI/ICIs) like cytotoxic T-lymphocyte associated protein 4 (CTLA-4) and programmed death-ligand 1 (PD-L1) antibodies are widely used but face limitations, including high cost, modest response rates, and side effects [17–22]. PD-L1 and CTLA-4 are protein receptors expressed on cancer cells and T-cells, respectively, that downregulate immune response against cancer cells. Alternatively, adoptive cell transfer techniques (e.g., Chimeric antigen receptor (CAR) T-cells, mesenchymal stem cells) genetically enhance immune function [23–26]. Since the advent of ICIs and CAR T-cell therapies, immunotherapy has become a strong alternative to conventional treatments. Current strategies focus on T-cell activation within adaptive immunity, yet tumors evade immune detection by exploiting checkpoints on antigen-presenting cells (APCs) including dendritic cells (DCs), macrophages, and monocytes. These APCs initiate inflammation and tissue repair. Cancer cells evade macrophage phagocytosis by releasing “do not eat me” signals through surface markers like PD-L1, Disialoganglioside 2 (GD2), Cluster of Differentiation 24 (CD24), Stanniocalcin-1 (STC-1), Cluster of Differentiation 47 (CD47), and β2-microglobulin (β2M), facilitating immune evasion and tumor growth [27, 28].

Due to rising interest in site-specific immunotherapy, this review emphasizes the brain, liver, and lungs for their unique immune profiles and relevance in metastasis. These organs are among the most frequent sites for both primary and metastatic tumors, and each presents distinct anatomical, physiological, and immunological challenges that greatly influence the design of targeted immunotherapies. The brain is protected by the highly selective BBB [29]; the liver contains fenestrated sinusoidal vasculature and exhibits high metabolic and immunotolerant capacity [30]; while the lung’s extensive alveolar-capillary network constantly balances immune activation and tolerance in response to environmental antigens [31]. These organs often host both primary and metastatic tumors and differ significantly in immunology from the brain’s immune privilege, the liver’s tolerance role, to the lung’s vascular, reactive environment [32]. Their complexity demands targeted immunotherapies that can bypass local immune suppression. Studying these organs helps refine spatially regulated strategies to guide next-generation treatments [33]. Immunotherapy has revolutionized cancer treatment, through approaches such as adoptive cell therapy, ICIs, and vaccines to enhance T- cell responses for tumor suppression [34, 35]. These strategies rely on APCs activating T-cells via damage-associated molecular patterns (DAMPs) released by stressed and dying cancer cells and recognition of tumor-associated antigens (TAAs) expressed in high levels on tumor cells [36]. Both DAMPs and TAAs are a group of molecules and proteins/peptides that act as danger signals for the immune cells to recognize and activate the immune response [37]. Professional APCs include B cells, macrophages, and DCs, process and present antigens using major histocompatibility complex (MHC)-II and co-stimulatory molecules [38–40]. MHCs are proteins on the surface of T-cells that present peptides. In the TME, DCs and macrophages take up antigen presentation [41, 42]. While macrophages are phagocytic, they don’t migrate to lymph nodes, often promoting resistance to therapies [43–45]. In contrast, DCs migrate to lymph nodes, activating robust T-cell responses [46–49]. Tumor-resident DCs have demonstrated anti-tumor activity [50–52]. However, fibroblasts and macrophages often promote tumorigenesis in TME, making their regulation crucial for effective immunotherapy [53–58]. Strategies to stimulate tumor remodeling are essential for improving cancer therapeutics via immunotherapy [59–61].

Recognizing the significance of APCs, it’s vital to examine how their roles differ across organs like the brain, liver, and lungs each presenting unique immune barriers. The brain, protected by the BBB a compact layer of endothelial cells covered by pericyte and astrocyte, limiting immune surveillance, thus posing challenge for effective immunotherapy. However, microglia and infiltrating DCs offer potential therapeutic targets [62]. In the liver, immune tolerance exists to manage gut-derived antigens, but tumors exploit this to suppress immune responses. Liver-resident macrophages (Kupffer cells) and tolerogenic DCs need reprogramming for anti-tumor immunity [63]. The lung, constantly exposed to airborne antigens, balances immunity and tolerance. Although vascularized and immune-rich, lung tumors establish suppressive environments [64]. Understanding these dynamics can help design strategies that overcome organ-specific immunosuppression [65]. While the promise of organ-targeted immunotherapy is immense, conventional approaches face limitations, primarily due to tumor immune evasion. Tumors undergo genetic and signaling pathway changes to avoid immune recognition [66]. A key shortcoming of traditional therapy is poor targeting, which has fueled interest in nanotechnology. Nanoparticles (NP/NPs) offer advantages such as prolonged circulation, tumor-specific accumulation, controlled drug release, TME modulation, and immune activation [67–72]. Hypoxic TME conditions caused by aberrant vasculature further promote immune suppression, leading to accumulation of myeloid-derived suppressor cells (MDSCs), regulatory T cells (Tregs), and immunosuppressive factors like Transforming growth factor beta (TGF-β) and vascular endothelial growth factor (VEGF) [73–75]. MDSCs are active neutrophils and monocytes from the myeloid lineage that expand under cancerous conditions. Tregs are immune cells that limit immune response upon exposure to foreign particles. NP-based immunotherapy offers a breakthrough in addressing these barriers. Beyond drug delivery, NPs can function as stimuli-responsive agents activated by pH, redox shifts, near-infrared (NIR) light, or magnetic fields. Various nanomaterials fulfill distinct needs: polymeric NPs offer controlled release and biodegradability [76], gold NPs enable photothermal therapy (PTT) and imaging [77], liposomes provide high biocompatibility [78], while microneedles [79], and scaffolds [80], enable localized delivery and sustained immune activation [81]. Other platforms like 2D nanosheets [82] and nanogels [83] also show potential in responsive and targeted cancer immunotherapy.

**Fig. 1:**
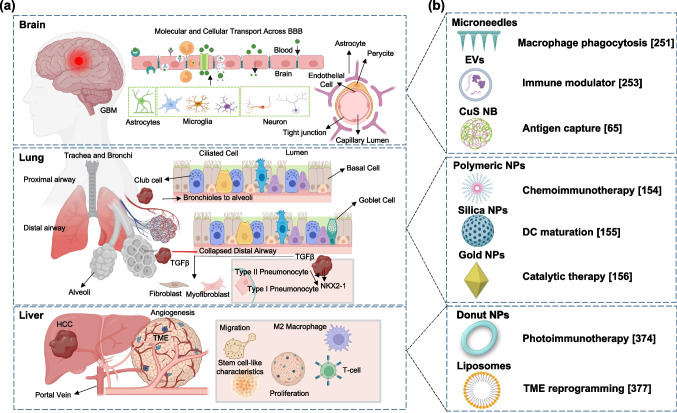
Representing the significance of nanotechnology and biomaterials in organ-specific immune modulation. **a** Schematic illustration of anatomical features and distinct TME in brain, lung, and liver cancer. **b** Immune system activating nanoplatforms include microneedles, extracellular vesicles, liposomes, polymeric, gold, and silica NPs

To highlight their clinical utility, explores NP-based immunotherapy across GBM, lung cancer, and HCC. For each, we outline the disease context, followed by recent nanotechnological approaches designed to overcome TME barriers and stimulate innate immunity for precise, effective treatment. Figure 1 illustrates the distinct features of TME in GBM, lung cancer, and HCC along with an overview of varied nanomaterials and 3D-biomaterials employed for the effective activation of immune response in cancer therapy. Figure 1a reveals TME in GBM, lung cancer, and HCC. In the brain, the scheme represents the structure of the BBB, which is primarily composed of endothelial cells surrounded by pericytes and astrocytes, forming a tight junction. The presence of the BBB hinders the entry of drugs and small molecules into the brain, making GBM therapy a major challenge to date. In the lungs, the schematics represent the entire respiratory system with trachea, bronchi, and alveoli. The illustration depicts the presence of various cells in the inner lining of the airways. This includes club cells, basal cells, goblet cells, and ciliated cells. The figures also show the involvement of NKX2-1 and TGF-β, which play active roles in tumor differentiation and the development of fibrosis, respectively, resulting in the collapse of the respiratory system. To illustrate the liver, the diagram depicts the development of HCC. In HCC, the TME is characterized by the presence of proliferating cancer cells, M2 macrophages, cancer cells with stem cell-like properties, and migrating cancer cells, which ultimately induce metastasis. Figure 1b represents various nanoplatforms and 3D-biomaterials that are widely studied for effective activation of innate immune response. Collectively, organ-specific NP platforms highlight how spatially targeted immunotherapy can reprogram the TME, stimulate immune responses, and improve treatment efficacy in hard-to-treat cancers.

## Lung carcinoma

Lung cancer is a disorder characterized by a dynamically structured microenvironment with a poor prognosis and high mortality rate globally due to inadequate diagnostic techniques and multiple subtypes posing a momentous health challenge worldwide [84–86]. Morphologically, lung carcinoma can be segregated into small cell lung cancer (SCLC) and non-small cell lung cancer (NSCLC), with the prevalence of cases accounting for 15–20% and 80–85%, respectively [87]. The major subtypes of NSCLC are adenocarcinoma, squamous cell carcinoma, and large cell carcinoma, with 40%, 25%, and 10% cases, respectively. WHO included the sub-classification of lung cancer in 2015 based on genetic profile [88].

NSCLC is characterized by multiple genetic mutations. Alterations in the epidermal growth factor receptor (EGFR) gene are quite prevalent, and individuals affected by this mutation benefit from tyrosine kinase inhibitors (TKI/TKIs), and one such popular TKI is osimertinib [89]. The rearrangements of anaplastic lymphoma kinase (ALK) in NSCLC are treated with alectinib, which has exhibited greater therapeutic efficiency in early stages of ALK-positive NSCLC patients [90]. KRAS-G12C alteration, which was for long considered untreatable, is now treated with sotorasib, and has drastically improved prognosis in comparison with docetaxel [91]. MET exon 14 skipping mutations are targeted with tepotinib [92].

SCLC is a aggressive form of lung cancer and is usually identified at later stages. Conventional therapy relies on platinum based chemotherapeutic compounds and developments have been made in immunotherapy [93]. The combination of ICIs such as durvalumab and atezolizumab with platinum-based chemotherapeutic drugs have improved survival rates [94]. Lurbinectedin a cancer therapeutic agent has received accelerated approval from Food and Drug Administration (FDA) for the treatment of metastatic SCLC [95].

Lung carcinoma possesses distinct immunological hindrances that influence the efficacy of cancer immunotherapy compared to other organs. The lungs are one such organ that is under constant exposure to inhaled particles, pathogens, and environmental toxins, which creates a well-balanced immune environment in order to facilitate immune tolerance [96]. This microenvironment comprises eminent levels of immunosuppressive cytokines (e.g., interleukin-10 (IL-10), TGF-β), Tregs, and MDSCs, which impair the activity and efficacy of cytotoxic T-cells and ICIs, respectively [97, 98].

Unlike immune-privileged organs like the brain, which physically restrict immune access via the blood–brain barrier, the lung allows immune cell infiltration. Still, it often skews immune responses toward tolerance and chronic inflammation [97]. Compared to the liver, which exerts systemic immunosuppression through tolerogenic antigen presentation, the lung’s barrier is more localized, acting within the tumor microenvironment. Furthermore, hypoxia, high tumor mutational burden, and abnormal vasculature in lung tumors create additional hurdles by promoting immune evasion and T-cell exhaustion [96].

Later stages of lung cancer are characterized by hypoxic TME which induce secretion of immunosuppressive TGF-β and interleukin-10 (IL-10) along with simultaneous enhancement of immune checkpoint molecules eg. PD-L1. This not only promotes cancer proliferation but also restricts the function of immune cells [96, 97]. In addition, Tregs and MDSCs further hinder anti-tumor immune response by forming a protective niche reducing immunotherapeutic efficiency of ICIs [98].

TMEs immunosuppressive nature can be further exacerbated by factors such as, tobacco smoking is one of the main contributors to lung cancer. Stage I and II lung cancer is usually curable, where the affected individual undergoes surgical resection, and patients with stage III lung cancer undergo chemotherapy and radiation therapy after surgery [99]. Individuals affected by lung cancer acquire resistance to radiotherapy and chemotherapy [100, 101]. The lung cancer’s stroma is complex and characterized by an active network of cells, constantly secreting cytokines and simultaneously remodelling the extracellular matrix (ECM), facilitating proliferation, drug resistance, and metastasis [102–104]. Thus, the stroma of the lung significantly affects the consequences of cancer therapy thus, conventional cancer therapy is inadequate to overcome the resistance mechanisms posed by stroma [105]. In spite of progress in research over the last decade, fighting lung cancer has been a major challenge, hence demanding a need for meticulously tailored remedial intervention to circumvent associated complexities [106, 107].

### Understanding immunogenicity, lung cancer microenvironment

Lung cancer cells produce various carcinogenic hallmarks that facilitate division, neovascularization, invasion, and subsequent spreading. The biological terrain of the lungs comprises endothelial cells, alveolar macrophages, smooth muscles, fibroblasts, and DCs [64]. The alveolar walls are lined by endothelial and smooth muscle cells facilitate immune cell migration and gas exchange. The proximal part of the lung comprises neuroendocrine cells, secretory club cells, undifferentiated basal cells, and mucus-producing cells, and alveolar type I and type II cells are found in the distal part [108, 109]. Macrophages maintain immunological homeostasis and control the progression of malignancy and inflammatory response [110, 111]. Chronic obstructive pulmonary disease raises the risk of lung carcinoma via mutation, remodelling of epithelial cells, and inflammatory mediators [112–114]. Ineffective activity of cytotoxic T-cells is an indicator of tumors due to their immunosuppressive microenvironment [115].

### Downregulated expression of PD-L1

The expression of PD-L1 has shown both favorable and unfavorable associations with ICIs due to limitations in gauging specificity, sensitivity, and selectivity in staining, slicing precision, and tumor heterogeneity [116–120]. Reports claim low and high expression of PD-L1 in the later and early stages of lung cancer, respectively [120–123]. However, greater expression of PD-L1 was observed on tumor-infiltrating T-cells [124]. Thus, PD-L1 may not be a reliable marker for determining the therapeutic efficacy in lung cancer. Upregulation of B7-H3 (B7 family immune checkpoint molecule) in the lung has been reported to facilitate pro-cancer and immunosuppressive functions, and CD47 blockade improves phagocytosis in lung cancer models [120, 125–127].

### Molecular transformation of HER2 in lung cancer

Human epidermal growth factor receptor 2 (HER2) belongs to the HER family, which in the absence of ligands forms heterodimers with other HER members [128, 129]. The high expression of HER2 activates downregulation of cancer signaling pathways PI3K-AKT, RAS-RAF-mER-ERK, and triggers phosphorylation of tyrosine kinase [130]. HER2 anomalies are characterized by high expression of protein and gene alteration. Alterations involve insertion into exons of tyrosine kinase by in-frame and non-frame alteration with major variation repeating 12 base-pair insertions resulting in A775_G776insYVMA alterations (YVMAins), and subsequently that of G776delinsLC, P780_Y781insGSP, and G776delinsVC [131, 132]. Apart from that, reports have also claimed the observance of point mutation G776C and L755S [133]. HER2 alterations resist pan-HER TKIs [134, 135]. This is due to the molecular reconfiguration of protein, enabling a shift in the drug binding site, hence preventing binding of TKIs [136].

### Absence of antigens hinder lung cancer immunotherapy

Downregulation of MHC class I and II by cancer cells reduces therapeutic effectiveness and is attributed to the absence of MHC-II transactivator (CIITA) [137]. The low expression of MHC-I, β2M, human leukocyte antigen (HLA-A, HLA-B, and HLA-C) decreases neoepitopes and recognition of the tumor by cytotoxic T-cells [138, 139]. This alteration in lung cancer has resulted in a defective antigen presentation mechanism which could be overcome by blocking chromatin restructuring controller enhancer of zeste homologue 2 (EZH2) [140].

### Nanomedicine in lung cancer therapy

While conventional therapeutic techniques involve segmentectomy, pneumonectomy (entire lung removal) and lobectomy (removal of a lung lobe) followed by radiation and chemotherapy to target localized and advanced stages, respectively, for lung cancer, the therapeutic efficiency is limited by off-targeting of drugs and further mutation of carcinoma to develop drug resistance [106, 141]. This is where nanomedicines can prove to stand out by reducing chances of off-targeting and reducing the requirement of injection of high doses of drugs to limit drug resistance, and effectively turn a cold tumor into a hot one for effective recognition by T-cells [142].

Nanotechnology is a branch of science dealing with nano-scaled materials for various applications [143]. In biomedical engineering, NPs have been widely exploited for diagnostics and as therapeutic agents [144–146]. Over the years, they have been extensively studied for cancer therapy, diagnostics, and theranostics by integration of physics, biology, and chemistry [147, 148]. Nanocarriers can be engineered to deliver therapeutic agents controlled release or in response to stimuli such as pH, enzymes, redox reactions, NIR, and magnetic fields. The NPs can be conjugated with targeting ligands and peptides for active targeting of lung cancer by binding to overexpressed EGFR and folate receptor [149]. In recent times, nanomedicine has evolved drastically and has shown potential for effective immunotherapy in lung carcinoma models [150–152]. Table 1 provides an overview over the material of nanomedicine, external excitation power source, and targeting mechanism employed in the recent past for lung cancer immunotherapy.

**Table 1:** Nanotherapeutic systems for lung cancer immunotherapy

Nanoparticles	Material	Cell Line/Tumor Model	External Energy Source	Targeting Mechanism	Description
NBTXR3	Hafnium oxide	Anti-PD1 resistant 344SQR	High and low dose radiation	Intratumoral Injection	Radiation-induced STING activation and immune checkpoint blockade improved the abscopal effect [153]
CDDP-NPs	Poly(L-glutamic acid)-graft-methoxy poly(ethylene glycol)	Lewis lung carcinoma cells (LCC)	Radiation	EPR effect	Cisplatin-enhanced radiation therapy amplifies Calreticulin translocation augmenting ICD [154]
UCMS@Pep-aPDL1-RB	Silica dioxide	LCC cells (metastatic model)	NIR-I	EPR effect	Photodynamic effect in combination with peptide vaccine and anti-PDL1 blockade activated immune response [155]
AP-HAI	Gold and silica	LLC cells (Subcutaneous cancer model)	NIR-I	EPR effect	PTT and PDT mediated TAAs release and DCs maturation. [156]
IO@FuDex	Iron oxide	4T1 cells (Metastatic model)	Magnetic field	Magnetic field aided navigation	Immune checkpoint blockade (anti-PD-L1) and T-cell activators (anti-CD3/anti-CD28) reduced lung metastasis [157]
CN	Purssian blue	B16F10 cells (Metastatic lung cancer)	Alternating magnetic field	Marginated target/EPR	Hyperthermia-mediated TAAs release captured by the catechol group on CN acting as an antigen reservoir for DCs maturation and ICD [158]
IO@MM	Purssian blue/iron oxide	CT26 cells (Metastatic lung cancer)	High-frequency magnetic field	Macrophage membrane coating guidance and EPR	Magnetothermia releases TAAs captured by the macrophage membrane, activates DCs, aiding in ICD [159]
ZCMP	Iron oxide	LCC cells	Alternating magnetic field	Nebulization (Inhalation)	Mild hyperthermia and ROS-mediated activation of kappa-B trigger M1 polarization, actuating upregulation of NKG2D in NK cells [160]
eCPMV	Cowpea mosaic virus	B16F10 cell (Metastatic lung cancer model)	None	Accumulation and activation of neutrophils in the lungs	Elevated production of Il-12p40, Il-1β, Ccl3 (MIP1-α), IL-6, and Tnf-α in macrophages and DCs, posing an immunostimulatory effect [161]
M@BCG	Raw264.7 macrophage membrane	LCC cells	None	Macrophage membrane-mediated targeting	BCG-mediated training of TAMs in combination with αPD1 for immune response [162]
IL-12 circRNA LNPs	Ionizable lipid	LCC and HKP1 cells	None	Intratracheal administration	Transfection of RNA encoding IL-12 augmented T-cell infiltration [163]
MIO@RBC	Iron oxide	B16F10 cells (Metastatic lung cancer model)	Alternating magnetic field	Phagocytosis and micro-pinocytosis by cancer cells	Hyperthermia-aided antigens and DAMPs release captured by MIO and delivery to the lymph node for T-cell activation [164]
LRT	Zn/Al hydroxide	LCC cells	None	Layered structure target stroma	CD47 and calcium inhibition, Calreticulin translocation facilitate DCs maturation and T-cell activation [165]
CAS	Cu MOF	B16F10 cells (Metastatic lung cancer model)	None	FA coating	CDT, CQ-mediated autophagy reflux inhibition and catechol aided antigen capture for enhanced infiltration of T-cells (CD4^+^ and CD8^+^) [166]
SPION-CCPMs	Super-paramagnetic iron oxide nanoparticles	LCC and Eml4-Alk tumor cells	None	Intratracheal administration	Iron loading facilitates activation of macrophages to produce cytokines, RNS, and ROS [167]
CLDCu	Chitosan and Cu^2+^	B16F10 cells (Metastatic lung cancer model)	None	Aerosolization (Inhalation)	Cuproptosis-mediated activation of cGAS–cGAS-STING pathway. [168]

### Radiation-induced immune response

Radioimmunotherapy is a strategy combining radiation and immunotherapy for enhanced therapeutic outcome. One such study includes the delivery of ICIs in combination with radionuclides to induce cytotoxicity by means of ionizing radiation target and treat tumor [100, 169]. NPs have been screened to analyse therapeutic efficiency by means of radiation. The basic principal being the use of NPs with higher atomic number or delivering radiosensitizers which enhances energy deposition at the targeted tumor site upon radiation thereby amplifying ROS production resulting in deoxyribose nucleic acid (DNA) damage [170, 171]. This results is release of DAMPs and TAAs resulting in anti-tumor immune response facilitating immunogenic cell death (ICD) [172].

In a study Hu et al. and team combined radiotherapy and immunotherapy for effective treatment of anti-PD1-resistant lung cancer. The study emphasized on delivery of hafnium oxide NPs named NBTXR3 a popular radioenhancer used to treat sarcoma. The lung cancer mouse models were created by injecting 344SQR cancer cells and then treating them with radioimmunotherapy by employing anti-PD-L1, anti-CTLA4 antibodies, and NBTXR3. The NPs were injected into the tumor for effective treatment. The treatment strategy employed consisted of 3 fractions of 12 Gy radiation. The results revealed a significant enhancement in therapeutics by lowering number of Tregs and significantly improving cytotoxic T-cell infiltration [153].

NPs have been shown to enhance the abscopal effect, a fascinating feature of radiotherapy. The abscopal effect refers antitumor effect on targeted cancer cells leading to systemic anticancer response, as a result, suppressing metastatic cancer [173]. The abscopal effect was exploited by Wang et al. and their research team, who utilized cisplatin, a chemotherapeutic agent, to improve radioimmunotherapy. In this study they demonstrated the systemic efficiency of cisplatin encapsulated into poly(l-glutamic acid)-graft-methoxy poly(ethylene glycol) termed CDDP-NPs in synergy with anti-PD-1. The results revealed that cisplatin enhanced ICD and secreted C-X-C Motif Chemokine Ligand 10 (CXCL10), as a consequence activating the innate immune cascade, followed by infiltration of cytotoxic T-cells. The nanoformulation CDDP-NPs effectively targeted and accumulated in LCC tumors via the EPR effect, as assessed through platinization of cellular DNA, which was drastically lower in the case of free cisplatin-treated groups. Thus, the study by Wang et al. revealed a significant stride towards lung carcinoma therapy by combining radiotherapy and immunotherapy in mice bearing Lewis lung tumor models [154].

### NIR activable and semiconductor NPs

With rising interest in stimuli-responsive NPs, NIR light-mediated cancer therapy has made substantial developments with semiconducting NPs and photosensitizers capable of PTT and photodynamic therapy (PDT) [174–176]. Semiconducting NPs comprise metal sulfides, chalcogenides, and metal oxides, which, upon further combining with other semiconducting materials or metals. This leads to the formation of heterojunction nanocatalysts with shortened band gaps, which facilitate electron transport from the valence band to the conduction band upon excitation by an external source of energy, such as NIR and ultrasound. Thus, capable of treating cancer by means of hyperthermia and production of reactive oxygen species (ROS) [177]. Moreover, combination therapy involving ICIs have shown promising results in suppressing tumors.

In the recent past, researchers have suggested the inhibition of PD-1/PD-L1 pathway to improve immunotherapeutic efficiency. To further investigate on the same Wang et al. and team [155] fabricated a up converting mesoporous silica NPs (UCMS) which were further loaded with photosensitisers and AL-9 peptide and PD-L1 inhibitor. UCMS exhibited accumulation into metastatic tumor via EPR effect signifying the NPs capability to passively target tumor. The proposed therapeutic system proved to be a potent strategy against metastatic Lewis murine lung carcinoma in spine by promoting ICD via enhanced effector T-cell infiltration [178]. Ou. et al. and research group [179] employed imatinib-loaded layer-by-layer PLGA NPs containing IR-780 and glucocorticoid-induced TNF receptor (GITR) antibody to target Tregs. The nanomedicine was experimented on lung cancer to scrutinize therapeutic efficiency. The results revealed substantial suppression of Tregs, and at the same time, photosensitizers facilitated both PTT and PDT, thereby aiding in presentation of antigens and synchronous maturation of DCs, resulting in infiltration of cytotoxic (CD8^+^) and helper (CD4^+^) T-cells.

**Fig. 2:**
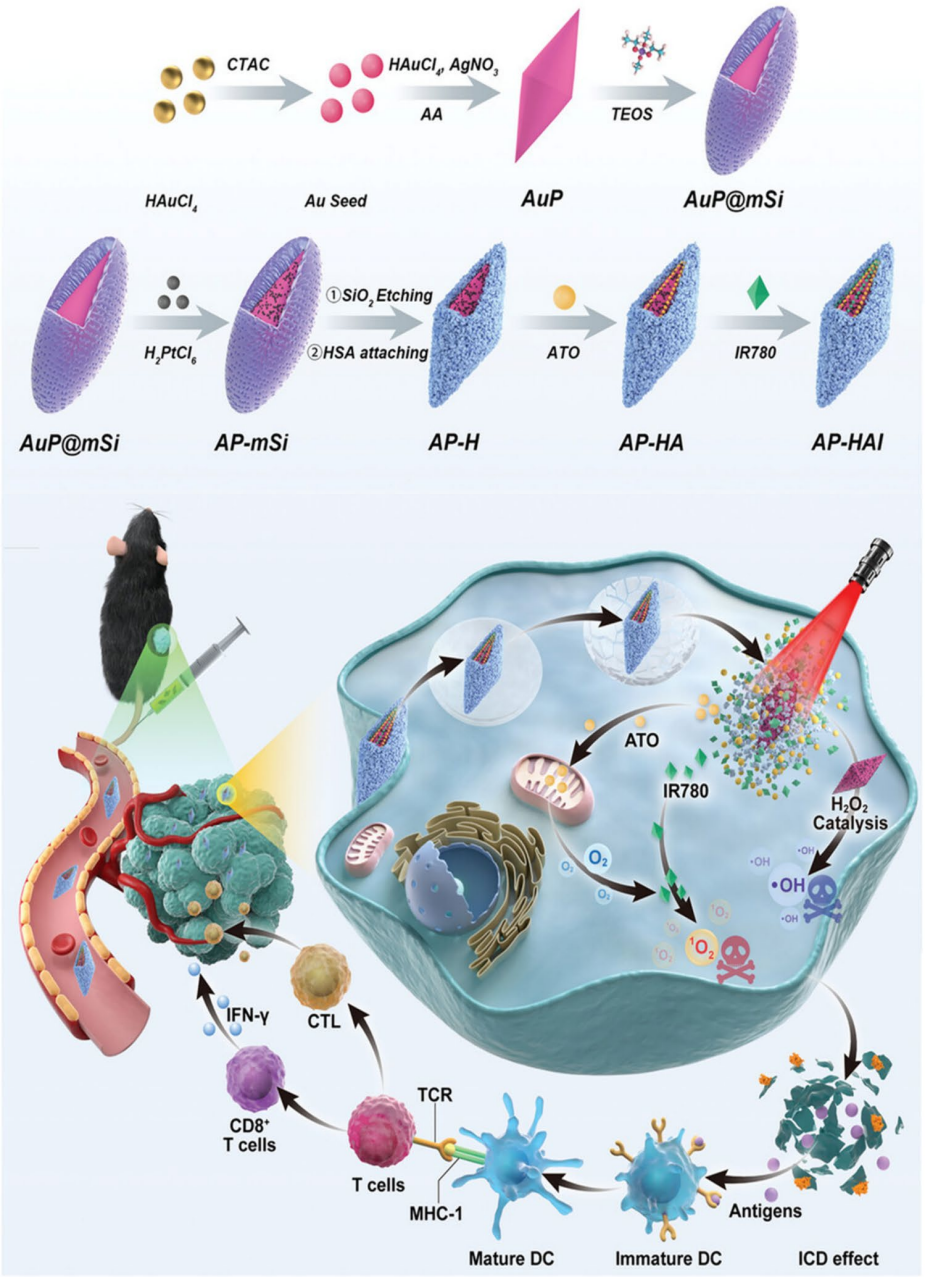
Synthesis of AP-HAI nanoprobes with Au seeds forming bioconical structure, AuP coated with silica and doped with platinum, followed by SiO2 etching to load atovaquone and PDT agent IR780. AP-HAI facilitates PTT and PDT, ATO disrupts respiration activating immune response [156]

In an intriguing study, Zhang et al. [156] synthesized a novel bipyramidal gold nanoparticle (AuP) with platinum nanozymes (nPt) on the surface **(**Fig. 2**)**. The ultrasmall nPt was grown controllably on the surface of AuP, which is mesoporous silica dioxide (SiO_2_). Modifying AuP with SiO_2_ and nPt is termed AP-mSi, with enhanced peroxidase-like activity and enabling the photothermal effect achievable by NIR (808 nm). The SiO_2_ template on AP-mSi was further removed and conjugated with human serum albumin (HSA) and loaded with atovaquone (ATO) and IR780 to obtain a theragnostic nanozyme probe (AP-HAI). The effectiveness of peroxidase-like activity exhibited by IR780, increased due to the blockade of tumor respiration metabolism aided by ATO [180, 181]. The nPt nanozyme on the outer surface produced hydroxyl radicals by catalyzing H_2_O_2_ [182]. The production of dual ROS (·OH and ^1^O_2_) by AP-HAI stressed the endoplasmic reticulum (ER) to translocate calreticulin (CRT) and high mobility group box 1 (HMGB1 – DAMPs molecule) release from cancer cells. This led to DCs maturation, facilitating the infiltration of 9.17% interferon-gamma (IFN-γ⁺/IFN-γ) cytotoxic CD8⁺ T cells into the lung tumor, a significantly higher percentage than the PBS control (0.3%). Moreover, the tumor temperatures elevated to 54.3 °C upon NIR irradiation, suggesting a strong accumulation of AP-HAI, which was also marked by tumor suppression with no harm to vital organs. Apart from posing a photothermic effect, AP-HAI facilitated NIR mediated real-time imaging of lung cancer cells due to its excellent capability to accumulate in the tumor. The application of thermal effect as a result of NIR irradiation was hypothesized to reduce interstitial fluid pressure and enhance the recruitment of immune-associated cells, leading to accelerating ICD.

### Magnetic field-responsive NPs

Magnetic NPs primarily comprise iron oxides, which have fascinating properties for effective lung cancer therapy. This class of NPs not only facilitates stimuli-responsive release of drug, but also offers activation of hyperthermia, mediate nanoparticle-induced extracellular leakiness (NanoEL), and imaging upon exposure to a magnetic field due to the paramagnetic nature of Fe [183, 184]. NanoEL refers to the induction of microgaps between adjacent endothelial cells by nanoparticles, allowing for enhanced accumulation of NPs in the absence of the EPR effect [11]. The magnetic field-responsive NPs can also be exploited for effective accumulation of nanomedicine at the tumor via magnetic field-guided accumulation [185, 186]. Hyperthermia induces apoptosis, thereby making the tumor susceptible to chemotherapeutic drugs, radiotherapy, and immunotherapy [187, 188].

In an interesting study, Chiang et al. reported the use of magnetic iron oxide NPs in combination with Fucodian and aldehyde-functionalized dextran (IO@FuDex^3^), which were further formulated with T-cell activator and ICIs [157]. IO@FuDex^3^ exhibited excellent targeting capability into tumor by means of an external magnetic field. The therapeutic system comprises of anti-PD-L1 and anti-CD3/CD28 to trigger the immune system. The results revealed a significant reduction in the number of metastatic nodules and prolonged mice survival rates in 4T1 metastatic lung cancer-bearing mice facilitated by infiltration of CD4^+^ and CD8^+^ cells which were activated due to initiation of tumor destruction at the primary site. Thus proving to not only eradicate primary tumor but also inhibit metastasis in lungs [189, 190].

**Fig. 3:**
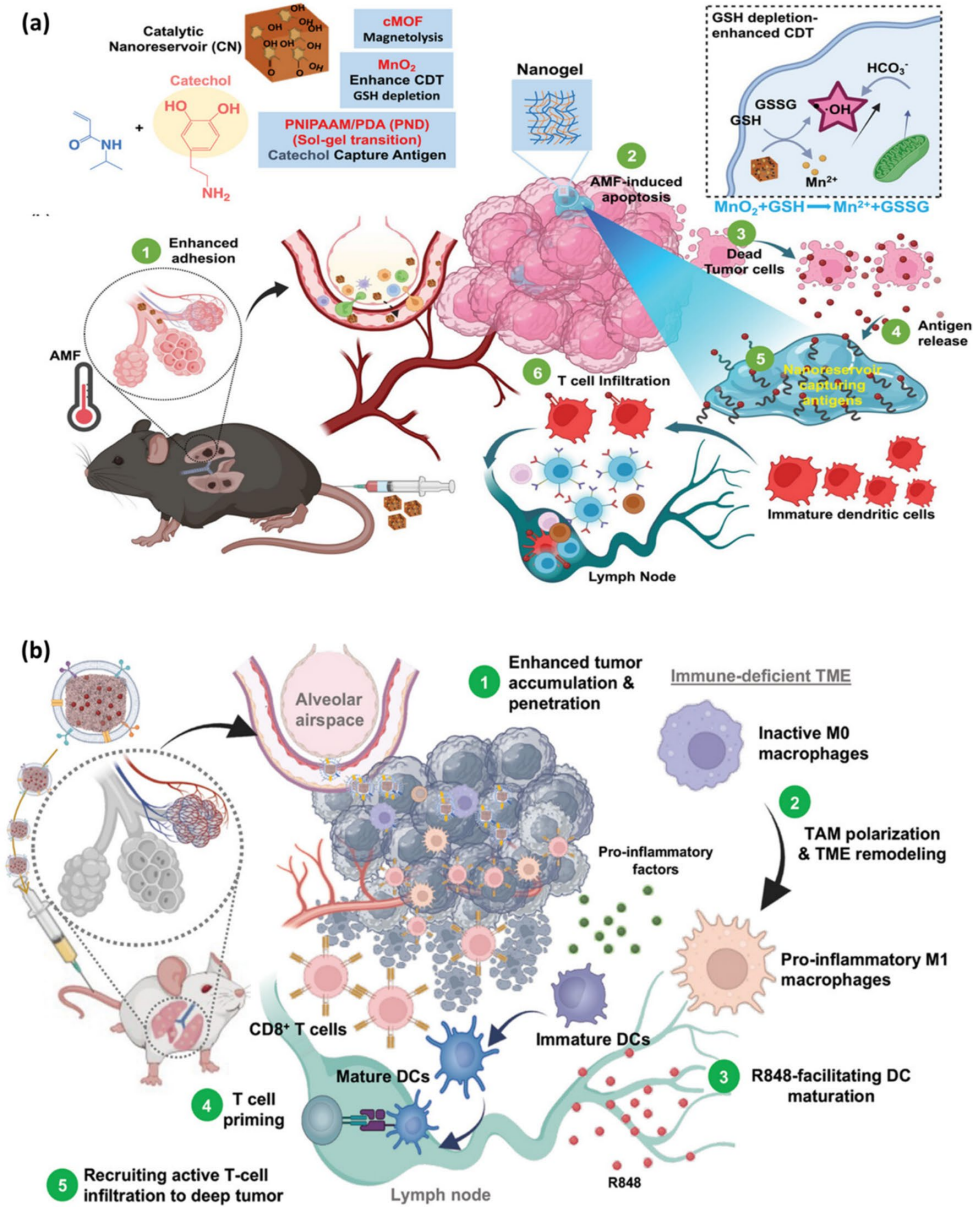
(**a**) Illustration of CN’s antigen adherence, acting as an antigen reservoir amplifying immunotherapy as a consequence of magnetothermal and redox chemodynamic effects facilitated by Mn2 + ions, promoting apoptosis [158]. **b** Schematic depiction of M1 macrophage membrane coated with IO@MM targeting lung metastasis and loaded with R848 to modulate immune response, turns cold tumour into hot tumour via hyperthermia for effective recognition by T-cells [159]

Recently, Yalamandala et al. [158] and co-workers came up with a hybrid nanogel termed catalytic nano-reservoir (CN) capable of capturing antigens **(**Fig. 3a**)**. The therapeutic nano-agent is composed of Prussian blue (PB) nanoparticle surface modified with poly (*N*-isopropylacrlamide)/polydopamine (PNIPAAM/PDA) and manganese oxide. PB after calcination exhibited a magnetothermal effect. Hyperthermia upon exposure to alternating magnetic field (AMF) and the chemodynamic effect induced by CN promoted apoptosis, leading to the release of TAAs. CN exhibited excellent targeting capability towards the lung via marginated targeting and uptake by phagocytic cells. The function of Mn in the design was to enhance the catalytic capability in the harsh TME [191, 192]. The catechol groups on the CN aided in capturing TAAs acting as a nano-reservior followed by the release of antigens for continuous stimulation of the innate immune system in BALB/c mice to inhibit metastasis via ICD [193, 194]. CN treated mice exhibited a significant reduction in tumor foci (< 10) upon comparision with control (~ 440 tumor foci). The tumor tissue revealed a threefold increase in infiltration of CD8^+^ T cell revealing a robust immune response. Moreover, the survival time in tumor bearing mice treated with CN + AMF + αPD-1 drastically increased accounting to ~ 60 days with a survival rate of 50%. The AFM activated CN proved to effectively treat orthotropic lung cancer in mice by selective targeting and hyperthermia to activate immune system by means of antigen capturing proving to be suitable for lung cancer therapy. Additionally, the presence of PNIPAAM/PDA gel on the surface assisted the release of chemoattractants and adjuvants for APCs [195–197]. The study demonstrated increased expression of inflammatory factors and cytokines such as IFN-γ, tumor necrosis factor-α (TNF-α), interleukin-12 (IL-12), and IL-10 which correlate with the maturation of DCs and T-cell infiltration [198, 199].

With tremendous efforts and focus put by researchers on maturation of DCs via NPs for stimulation of immune response, Wang et al. [159] and colleagues fabricated tumor-targeting NPs capable of programming tumor-associated macrophages (TAMs) into M1 macrophage to restore immune response at the TME **(**Fig. 3b**)**. TAMs are macrophages, a type of cell associated with the immune system that promote proliferation of solid tumors via expression and production of hypoxia-inducible factor 1 alpha (HIF-1α) and VEGF [200]. PB was synthesized by hydrothermal reaction and oxidized in the presence of atmospheric air to obtain porous magnetic iron oxide nanoparticles (IONPs). The IONPs were modified with M1 macrophages obtained by polarizing mouse macrophage RAW264.7 and named the lung metastatic colorectal cancer (CRC) treatment nanoagent as IO@MM. The macrophage membrane on IO@MM consisted of essential surface protiens upon comparision with isolated M1 macrophage membrane. This empowered IO@MM with targeting capabilities to selectively accumulate in metastatic lung cancer. The nanomedicine IO@MM was also loaded with resiquimod, a toll-like receptor 7 and 8 (TLR7/8) agonist [201, 202]. The therapy was combined with a high-frequency magnetic field (HFMF) to exploit the magnetothermal properties of IO@MM in the CRC mouse model, thereby converting cold tumors into hot tumors. The in-vitro results revealed greater cellular uptake for macrophage membrane-coated IO (IO@MM) when compared with uncoated IO where IO@MM treated groups revealed significant peak shift as per the flow cytometry data, thereby suggesting rapid endocytosis. The IO@MM nanomaterial loaded with doxorubicin (DOX) was studied for biodistribution, the results revealed significant accumulation in lungs thereby proving effective in evading immune clearance. Tumor bearing mice treated with IO@MM in combination with resiquimod (R848) revealed a drastic enhancement in CD8^+^ and CD4^+^ accounting to 34.18 and 55.19 respectively along with reduction in tumor nodules. The study proposed a unique strategy called nanoparticle-induced endothelial leakage to improve tumor leakage [203–205]. Hyperthermia promotes the release of heat shock proteins (HSP/HSPs), including neoantigens and DAMPs, especially HMGB1 and CRT, which were captured by macrophage membrane, to enhance inflammation and activation of lymphocytes [206–209].

Though researchers have experimented to exploit hyperthermia to its fullest, in a latest study, Chen et al. [160] introduced ZCMP nanomedicine with iron oxide as the base, which was further modified with Zn, CO, Mn, and polyethylene glycol (PEG) to obtain a mild magnetothermal innate immunity activator. Nebulization to deliver ZCMPs maximized its accumulation into lung lesions resulting in release of Fe ions under acidic conditions, and simultaneous mild magnetic hyperthermia produced ROS to selectively target and induce stress in lung cancer and modulate immune environment. The generated ROS activated nuclear factor kappa-B polarized alveolar macrophages (AM) to form the M1 phenotype, which released inflammatory molecules interleukin-1 beta (IL-1β), Interleukin-15(IL-15), and Interferon-beta (IFN-β). This promoted the stimulation of STAT5, c-jun, and GZMB, resulting in natural killer (NK) cells'proliferation and enhanced cytotoxic activity of NK cells by upregulating expression of Natural Killer Group 2D (NKG2D).

### Biological components in immunotherapy

NPs encapsulating biological materials have brought in a revolution in cancer immunotherapy. This includes the delivery of viral/bacterial components and nucleic acids via polymers and liposomes to minimize off-targeting and immune-associated complications. Nanocarriers loaded with ribonucleic acid (RNA), cytokines, and adjuvants have been reported to activate T-cells and their infiltration by enhancing antigen expression in APCs [210, 211]. Apart from that, CRISPR/Cas9 has also gained significant interest and has enabled a new path for gene editing in both cancer and immune cells [212]. This has aided in overcoming resistance mechanisms in cancer and enhanced immunogenicity. The successful encapsulation of biological molecules has gained interest over the years with positive results in lung cancer immunotherapy has been discussed in this section.

Viral vectors have been extensively used in with nanomedicine as a potent tool for cancer immunotherapy. Lizotte et al. reported inhalable virus-like NPs where the research group utilized Cowpea Mosaic Virus as an immunostimulant to trigger neutrophils at the tumor region [161]. An adenovirus-inspired system for the effective release of small interfering RNA (siRNA) into endosomes were designed by Feldmann et al. and team, where they knocked down tumor genes and inhibited tumor progression [213].

Apart from viral components, cellular materials such as cell membrane have also been vastly exploited to encapsulate therapeutic materials and NPs for effective and targeted delivery. In one such similar but unique attempt, Zhang et al. [162] employed macrophage membrane-entrapped Bacillus Calmette-Guérin (BCG) to induce trained immunity in TAMs and evade elimination. Cell membrane-based delivery systems are endowed with tremendous biocompatibility, tunability, and tumor targeting [214, 215]. Camouflaging of BCG with macrophage membranes apart from acting as vehicles not only prohibited the elimination of BCG biologics from the immune system but also enabled targeting of LCC bearing mice making it an ideal candidate for lung cancer intervention [216, 217]. Macrophage membrane enhanced internalization into the cell when compared with bacteria alone [218], which is beneficial in activating NOD2 signaling cascade thereby commencing trained immunity. The study revealed the superior targeting capability of M@BCG for the orthotropic LCC model. Remodeling of the tumor immune microenvironment was assessed by transcriptome and metabolomics analysis which revealed enhanced cytokines synthesis, restoration of glycolysis, and amino acid metabolism. They also revealed an increased expression of MHC II in M@BCG treated groups to promote polarization of bone-marrow-derived macrophages (BMDMs) to M1 macrophages [218, 219]. The combination group treated with M@BCG + αPD1 exhibited a significant reduction in tumor cells and inhibited proliferation as observed through hematoxylin and eosin (H&E) and Keil 67 (Ki67) staining. The study by Zhang et al. and team demonstrated successful accumulation of M@BCG into lungs and training of TAMs by wrapping BCG with macrophage membrane. Restimulation of lipopolysaccharides augmented production of interleukins-6 (IL-6) and TNF-α thereby proving to inculcate trained immunity. Moreover, combining ICIs like αPD1 with M@BCG significantly reduced tumor volume and restored the anatomical structure in LCC-affected mice by reprogramming immune landscape and exhibiting a long-lasting immune memory in TAMs proving to be suitable for lung cancer immunotherapy.

**Fig. 4:**
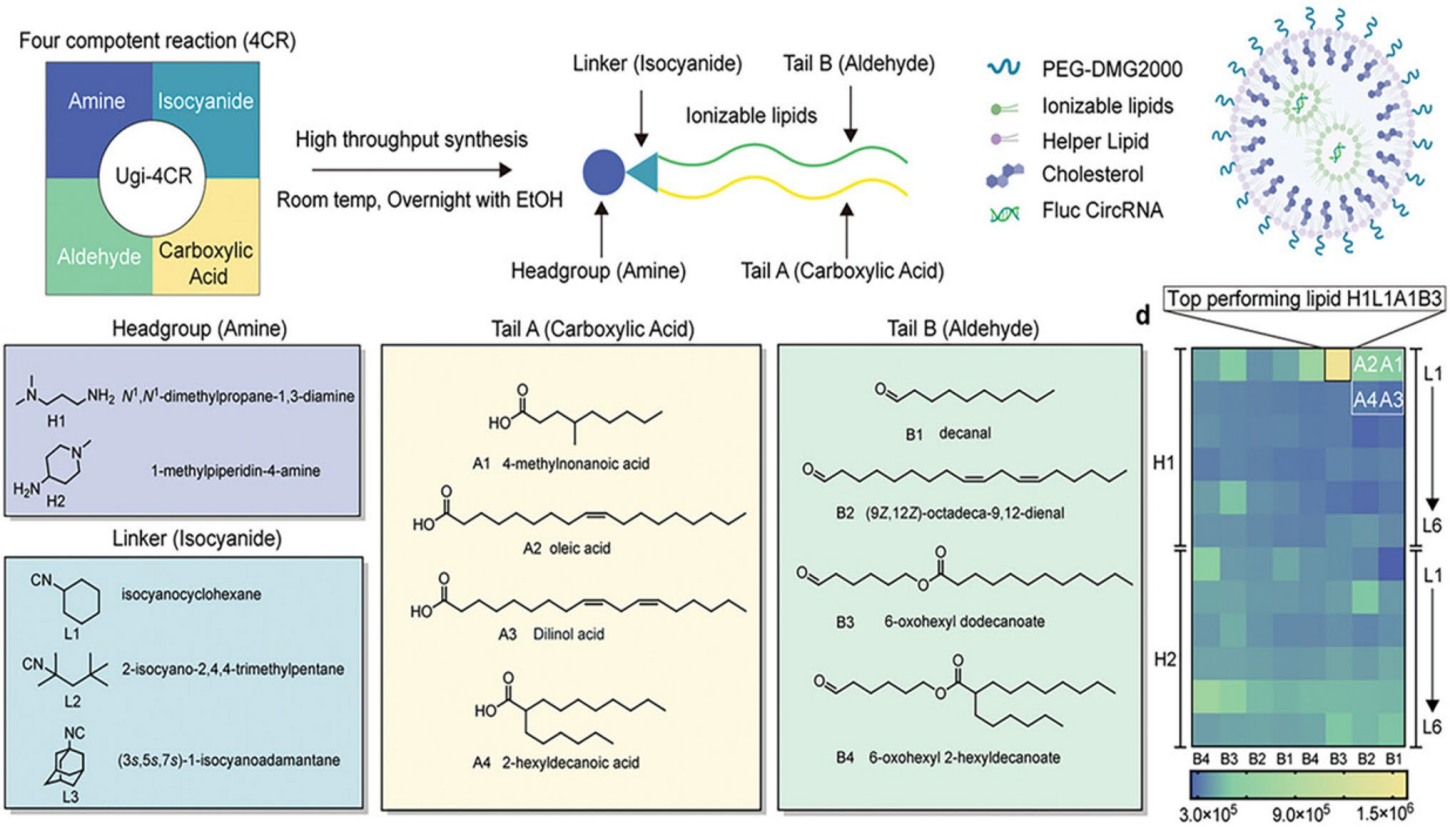
Schematic illustration of the design and fabrication of lipid nanosystems to deliver circRNA. The diagram represents the Ugi four-component reaction employed for the development of ionizable NPs by combining isocyanides, aldehydes, amines, and carboxylic acids, encapsulated with circRNA for tumour immunogenicity [163]

With researchers engaging in biological materials owing to its biocompatibility and targeting capability, there have been hybrid nanosystems comprising both synthetic and biological materials. In one such study, Xu et al. [163] employed ionizable lipid NPs (LNPs) encapsulated with circular RNA (circRNA) encoding IL-12 to induce a robust immune response for the treatment of lung cancer **(**Fig. 4**)**. The proposed lung cancer intervention showed notable transfection efficiency, accounting for four times higher than industrial-grade LNP. The therapeutic system was named H1L1A1B3 LNPs, which activated the NF-κB/IRF signalling pathway in turn leading to infiltration of immune cells to facilitate tumor suppression in LLC and HKP1 lung cancer mice models. Intratracheal administration ensures targeted delivery and reduced possibilities of off-targeting. The delivery of IL-12 circRNA directly to lungs significantly reduced systemic toxicity and aided in tumor regression via activating NF-κB and IRF pathways, augmenting CD8⁺ T-cell infiltration.

Another classic example of a hybrid nanosystem involves a novel study, where Huynh et al. and colleagues reported hitchhiking of multi-grained iron oxide NPs facilitated by red blood cells and named the therapeutic system MIO@RBC [164]. The hitchhiking capability of the proposed system not only facilitated immune evasion of nanostructures but also enhanced circulation time and accumulation of nanostructures into the metastatic lung cancer model [220, 221]. The magnetic field-responsive iron oxide NPs induced apoptosis via hyperthermia and simultaneously aided in the capture of TAAs to facilitate DCs maturation, followed by cytotoxic T-cell infiltration.

### Metal-based nanomedicine for lung cancer immunotherapy

The following section focuses on metal based NPs employed for delivering lung cancer interventions and functions of metallic ions and NPs capability to induce apoptosis and immune response with internal stimuli. Metal ions pose significant hindrances to cancer proliferation by means of ROS generation and evoking innate adaptive immunity. The same has been discussed in detail on the mechanisms scrutinized for effective cancer immunotherapy.

**Fig. 5:**
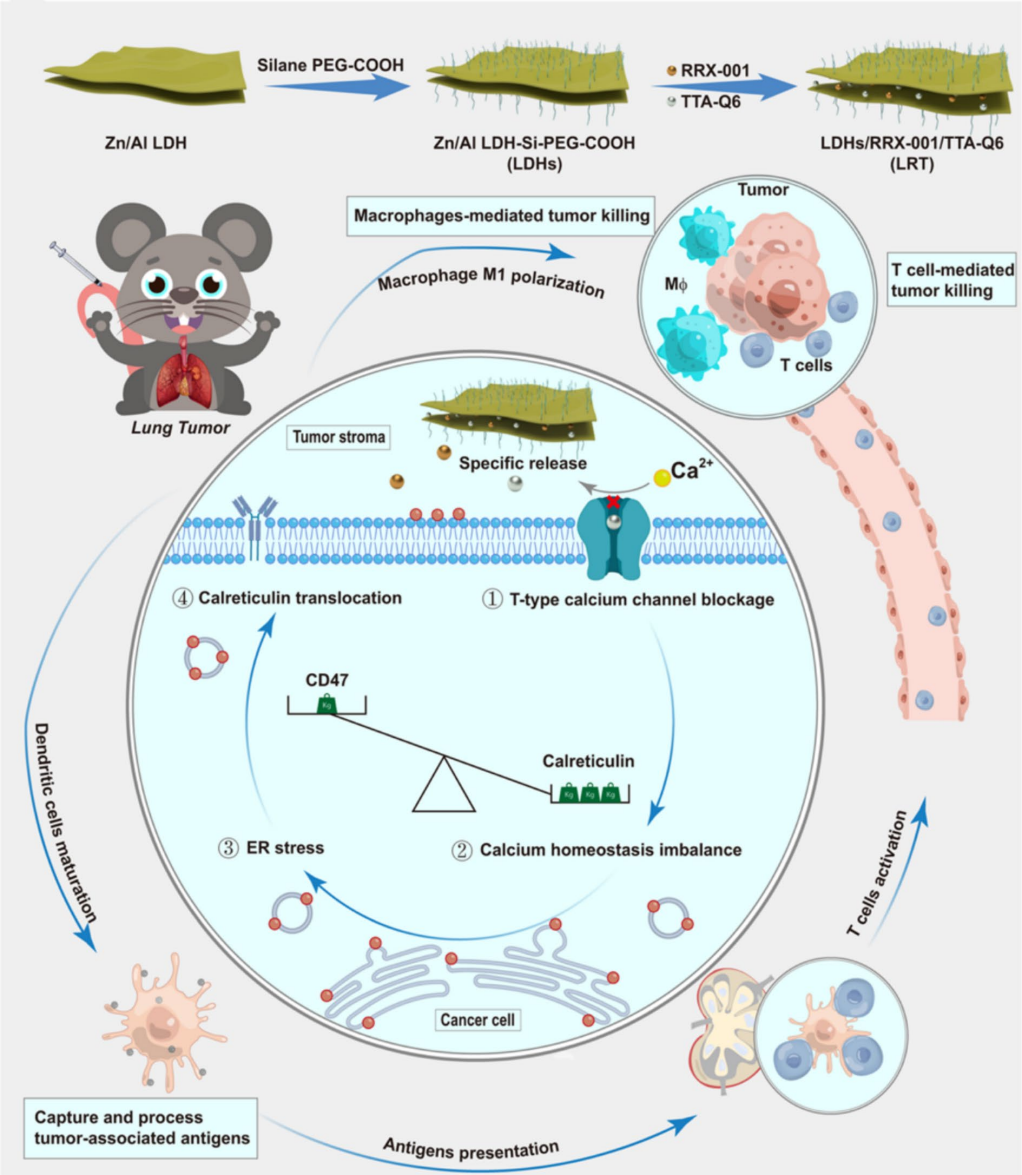
Illustration of LDH fabrication and subsequent loading of RRX-001 and TTA-Q6 to obtain LRT nanosheet. TTA-Q6 facilitates blocking of calcium influx, resulting in ER stress in parallel with downregulation of CD47. ER stress-mediated translocation of CRT enables polarization to M1 macrophages and maturation of DCs via capturing of TAAs [165]

In an interesting study involving metal based nano sheets, Guo et al. [165] and team designed an immunotherapeutic nanomedicine for orthotropic lung cancer with a sub-micro-sized double hydroxide Zn-Al (LDH) as a carrier for delivering RRX-001 and TAA-Q6 **(**Fig. 5**)**. The co-delivery of RRX-001 and TAA-Q6 facilitates the inhibition of CD47 and calcium channels, respectively. The inclusion of small molecular drugs triggers the antitumor effect by TAMs, which activates the maturation of naive DCs, followed by T-cell infiltration mediated by antigen-presenting DCs. They utilized an acid-responsive LDH for loading RRX-001 and TTA-Q6 and termed the nanoparticle as LRT. The sheet shape and micro size of LDH permitted the accumulation of LRT into the tumor stroma. This enabled the entry of TTA-Q6 into calcium channels via intracellular spaces, thereby inhibiting calcium ion uptake by tumor cells, promoting ER stress and calcium deficiency in tumor cells [222, 223]. This results in the relocation of CRT into the plasma membrane [224, 225]. Subsequently, RRX-001 aids in the negative regulation of CD47 proteins [226, 227]. The combination therapy aids in an anti-tumor effect and DCs maturation, mediated T-cells training and ICD. ER stress led to the release of CRT into the plasma membrane, which was assessed by immunofluorescence staining and gauged by flow cytometry and confocal microscopy. Significant activation of mature DCs (CD86) was observed in orthotopic mice models treated with LRT, which exhibited a differentiation and presentation of T-cells (CD4^+^ and CD8^+^) in the spleen, comprising effector memory (T_EM_, CD62L^−^CD44^+^) and central memory (T_CM_, CD62L^+^CD44^+^) T-cells. The downregulation of CD47 in tumors inhibited immune evasion by decreasing the interaction between CD47 and signal regulatory protein α (SIRPα), aiding in phagocytosis by macrophages [228, 229]. Moreover, LRT-treated groups exhibited a survival rate of 83% along with a significant increase in lung volume as obtained through 3D reconstruction of lungs.

Another study involving metal based NPs include involvement of Fenton-like reaction for the induction of apoptosis. Chiang et al. [166] and colleagues developed a copper (Cu) based metal–organic framework (MOF/MOFs) with antigen-capturing capability for an effective and robust immune response. The nanogel system consists of chloroquine (CQ) loaded into porous MOF and coated with PDA to facilitate antigen capture to induce an immune response and effective cancer therapy [195, 197, 230]. It was further modified with folic acid (FA) to enable active targeting of lung metastasis and named the nanomedicine as CAS. The MOF, consisting of the Cu central atom, was held by a tritopic organic linker 1,3,5 benzenetricarboxylic acid (BTC), and comprised of a coordinate covalent bond, which is susceptible to low pH as a result of releasing Cu ions upon exposure to acidic TME. The Cu ions facilitated Fenton-like reaction by breaking intracellular hydrogen peroxide (H_2_O_2_) to produce hydroxyl radicals, thereby inducing oxidative stress in cells. The function of CQ was to inhibit autophagy by preventing lysosomal fusion with autophagosomes [231]. Autophagy is a mechanism employed by cells to maintain cellular health. The inhibition of autophagic flux was affirmed by LC3B staining, which revealed a notable accumulation of autophagosomes in cancer cells. The use of CQ not only enhanced the efficiency of chemodynamic therapy (CDT) but also enabled apoptotic cell death [232]. They also combined CAS with anti-PD1 to study for a possible chance of improvement in immunotherapeutic efficiency, and the result showed satisfactory results with a significant reduction in the number of pulmonary metastatic nodules. Moreover, the presence of catechol groups on the surface of CAS facilitated the capturing of DAMPs and neoantigens such as CRT, HSPs, actinin-4 (Actn4), ribosomal protein L13a (Rp113a), and elongation factor 2 (Eef2) [233–236]. Thereby, marginally enhanced infiltration of T-cells (CD4^+^ and CD8^+^) subsequently leading to ICD. Though the Cu based CAS proposed by Chiang et al. exhibited immune response the same may not hold true for other metal based NPs for an effective activation of immune system.

T-cell being the primary agents for destroying cancer cells are difficult to be activated and meager activation would be ineffective in treating cancer. Thus, researchers have been experimenting with combination therapy involving metal ions and inhibitory molecules. In one such study, Horvat et al. and team came up with a conventionally unusual application of super-paramagnetic iron oxide nanoparticles (SPIONS). The SPIONS were trapped in the copolymers of polysarcosine and poly(S-ethylsulfonyl-l-cysteine) via crosslinking to obtain SPION-CCPM. The proposed study revealed SPIONS-CCPMs capability to trigger TAMs for the production of cytokines and reactive nitrogen species (RNS) with remarkable anti-tumor activity via iron loading. They also validated the remodelling of the immunosuppressive lung cancer model marked by infiltration of CD8^+^ after treatment with cirotonib a TKI molecule [167].

With innovative strategies being employed for robust immune response, reduction of Cu^2+^ ion is one such effective stratergy to induce cuproptosis. In a recent interesting study, Yan et al. constructed an inhalable nanodevice termed CLDCu for effective lung cancer therapy. CLDCu was designed to overcome the challenges associated with cupperoptosis with disulfiram loaded heparin-tocopherol succinate core and Cu^2+^ ions containing chitosan shell for effective accumulation in lung lesions. Both disulfiram and Cu^2+^ ions released from CLDCu nanodevice upon exposure to the acidic pH of lung carcinoma and formed a complex termed CuET, which inhibited the efflux of protein ATP7 B, leading to robust cuproptosis and STING pathway. The activation of the immune response was affirmed by DCs maturation and the simultaneous administration of anti-PD-L1 in lung cancer mouse models, activated significantly boosted survival, accounting for 57% [168].

## Glioblastoma

GBM is one of the most aggressive and treatment-resistant brain tumors, presenting major challenges in clinical management [237]. Despite advances in surgical resection, radiotherapy, and chemotherapy, the prognosis for GBM patients remains poor, underlining the urgent need for advanced therapeutic strategies [238]. Critical challenges continue to hinder effective GBM treatment. The highly infiltrative nature of GBM cells makes complete surgical resection nearly impossible, leading to rapid recurrence and treatment failure [239].

Heterogeneity in GBM associated with cellular and molecular levels hinders targeted therapy, as subpopulations within tumor exhibit variable treatment responses [240]. Another key challenge is the immunosuppressive TME comprising MDSCs and TAMs which secrete IL-6 and TGF-β, which allows tumor evasion from immune surveillance and limits the efficacy of immunotherapies by facilitating T-cell exhaustion [241]. GBM impedes antigen presentation and immune activation by decreasing MHC expression [242].

Apart from the mentioned complications, GBM is encompassed by unique barriers to immunotherapy compared to other vital organs, mainly due to the brain's immune-privileged nature. The BBB is one of the significant obstacles that limit the infiltration of immune cells and hinder the delivery of immunotherapeutic agents to the brain [240]. Even when the BBB is disturbed in GBM, immune cell trafficking remains limited. Additionally, the brain's microenvironment is characterized as anti-inflammatory, with low levels of APCs and elevated levels of immunosuppressive factors, such as TGF-β and PD-L1 [241]. Unlike organs like the liver, which systemically suppress immune responses, the brain imposes local immune restriction. In contrast to the lung, though immune cells exist, they are usually dysfunctional, and the brain largely dismisses immune surveillance altogether. Leading to reduced efficiency of ICIs and adoptive cell therapies [242]. Moreover, the presence of BBB comprising of endothelial cells covered by pericytes and astrocytes form a tight junction inhibiting penetration of therapeutic molecules [8]. Thus, the distinct physical and immunological barriers in the brain require specialized strategies to enhance immunotherapy response.

Efforts to reprogram the TME, such as ICIs, myeloid-targeting therapies, and tumor vaccines, have displayed promise but require further optimization to counteract the complex interplay of immune-suppressive factors in GBM [243].

### Challenges associated with GBM therapy

The presence of the BBB remains a difficult obstacle, restricting the delivery of many effective therapeutics to the tumor site. While strategies such as focused ultrasound, convection-enhanced delivery (CED), and NP-based systems have been explored to enhance BBB permeability, their clinical translation remains limited due to safety concerns and inconsistent efficacy [244, 245]. Furthermore, therapy-induced resistance, driven by adaptive molecular alterations and tumor plasticity, additional compromises long-term treatment success [246]. Addressing these concerns requires a multifaceted method that integrates progresses in nanomedicine, immune engineering, and precision oncology to improve more effective and strong GBM treatments [247].

### Nanotechnology in GBM immunotherapy

Over the past decade, researchers have revealed promising outcomes for GBM immunotherapy via drugs with significant advancements and positive results in the early clinical stage, but theories have proved to be futile in later clinical stages. The cause of ineffective results in immunotherapy of GBM via immunomodulatory drugs can be attributed to the presence of the BBB, plasticity, systemic toxicity, heterogeneity, immunosuppressive TME, and autoimmune reactions [248]. Moreover, the conventional technique involving surgical resection of GBM is limited by partial removal of tumor where a small part at the edge is left out due to the interaction of tumor with healthy brain tissue [245]. Thus, to overcome these interruptions, nanoplatforms have attained significant interest and evolved over the decades for the effective delivery of molecules and therapeutic agents due to properties such as tunability, cargo loading, BBB penetrability, and deep tumor penetration [72]. Various nanomedicines have been widely studied, and some have been marketed for real-time use, which predominantly include liposomes, polymeric and metallic nanomedicines, and extracellular vesicles (EV/EVs). In the sections ahead, we have summarized and discussed various potential nanoplatforms in recent times for effective GBM immunotherapy under various classifications. Table 2 describes various nanoparticle based therapeutic models that were severely scrutinized to reveal their GBM suppressive capabilities and their use cases.

**Table 2:** Therapeutic systems for GBM immunotherapy

Biomaterials and Nanoparticles	Therapeutic Material	Cell Line/Tumor Model	External Energy Source	Administration Technique	Description
CQ@CAS	Fe MOF loaded with CQ and coated on scaffold	ALTS1C1 cell (Post-operative GBM model)	None	Post-surgical placement of a scaffold in the resected cavity	Fenton-like reaction, autophagy reflux, and 3D printed elastomer acts like an antigen for sustained ICD. [249]
GMAN@CMN	Oncostatin blocking NPs loaded in microneedle array	Luci^+^GL261 cells	None	Post-surgical placement of microneedle array	Anti-siglec 10 and siRNA delivery trigger macrophage-aided phagocytosis [250]
CAR-neutrophil@R-SiO_2_-TPZ NPs	Tirapazamine-loaded silica dioxide	Luciferase-expressing GBM cells	None	Intravenous injection	Chemotherapy and CAR-neutrophil mediated tumor cell death [251]
EVs@dox/sgCD47/IL-9	Doxorubicin loaded EVs	GL261 cells and GL261-Luc cells	None	Intravenous injection	Chemotherapy, overexpression, and downregulation of IL-9 and CD47, respectively, activating ICD. [252]
RVG@GY	Gold nano-yarns delivering palbociclib-loaded dendrimer	ALTS1C1	Alternating magnetic field	Intravenous injection	CDK inhibitor and dendrimer activate T-cell recruitment, eddy current disrupts cell–cell interaction at the tumor site for T-cell penetration [253]
OT@COF-RVG	Temozolomide and OSMI-4 loaded COF	U87MG cells and GL261 cells	LED (White) light	Intravenous injection	White light/ESIPT mediated ROS generation, and chemotherapy releases DAMPs, which infiltrate T-cells. [254]
CuS NB	mPEG-b-C18 encapsulating copper sulphide nanoflakes	ALTS1C1 cells	NIR-II	CED	NIR-II mediated PTT and membrane disruption polymer release of TAAs captured by amine groups for sustained ICD [255]
CM-PNPs	Piezoelectric NPs	U87 MG cells	Low-intensity pulsed ultrasound	Intravenously infused	Ultrasound stimulation produces inflammatory cytokines and ROS, activating the immune response. [256]

### Implantable 3D biomaterials and scaffolds

Localized and implantable drug delivery systems further address brain targeting challenges by avoiding systemic circulation barriers and safeguarding sustained drug release at the tumor site. CED is one method that smooths the direct infusion of NPs into tumor cells, increasing drug distribution within the brain and improving local treatment efficacy [257]. CED refers to delivering nanoparticles or therapeutic drugs directly into the brain via bulk flow through a catheter to the desired region in brain [258]. Thermosensitive hydrogels loaded with immune-modulating agents and PTT NPs also provide a platform for repeated local treatments. These hydrogels allow sustained and controlled drug release, while simultaneously enhancing immune activation within the TME to support long-term anti-tumor immunity [259].

**Fig. 6:**
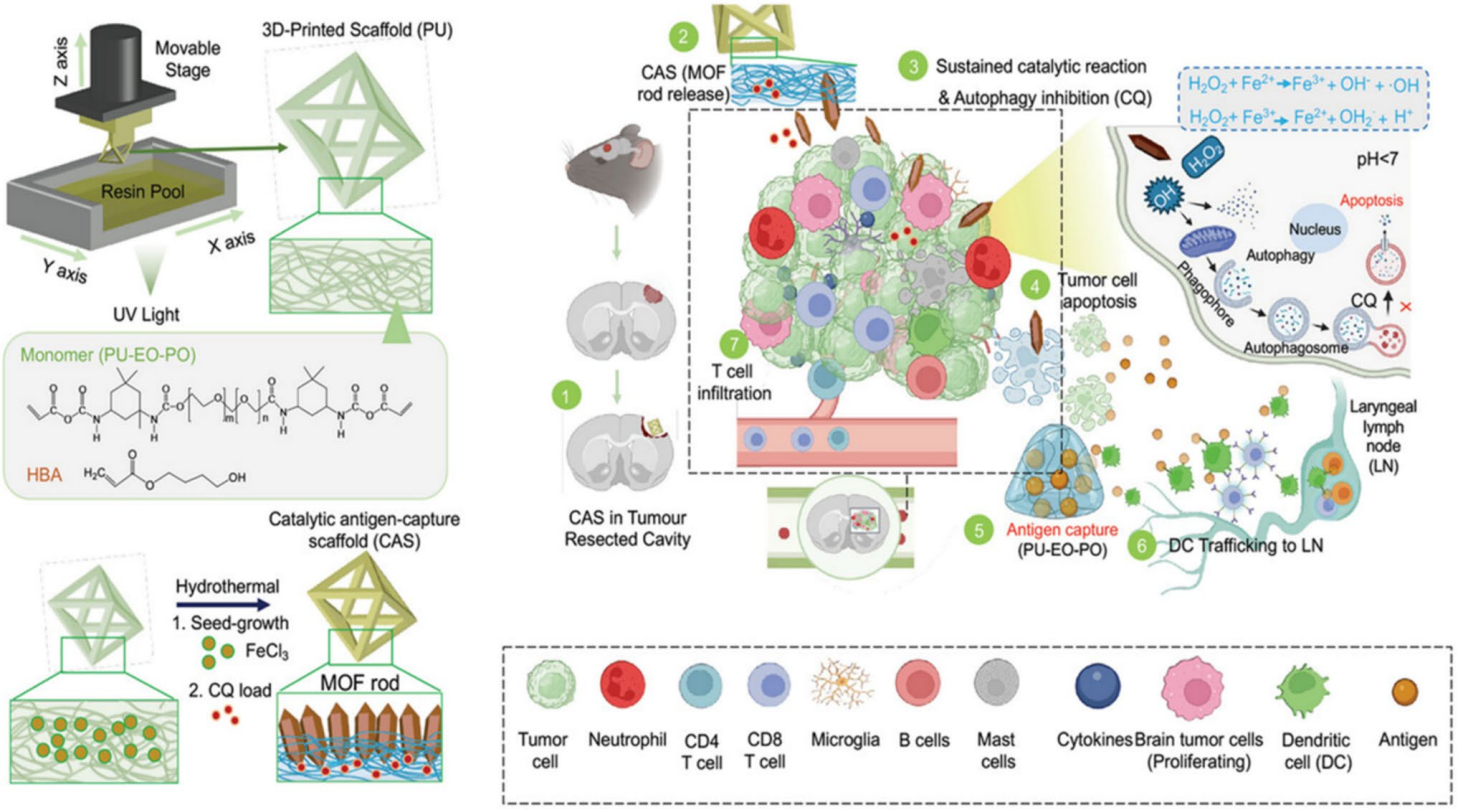
Scheme represents fabrication of CAS via 3D-printing followed by coating and loading with MOFs and CQ for catalytic activity and inhibition of autophagy, respectively. CAS was implanted post-GBM resection to initiate Fenton-like reaction and host antigen to enhance GBM immunotherapy [249]

An additional promising development in GBM therapy comes from biomaterial scaffolds engineered for post-surgical treatment. Scaffolds are polymeric matrices usually composed of drugs and growth factors that not only release therapeutic molecules but also act as a supporting structure for cells to penetrate, reside, and proliferate. Yalamandala et al. [249] developed a localized, post-surgical intervention using a 3D-printed elastomer scaffold, designated CQ@CAS, aimed at enhancing the treatment of GBM following tumor resection **(**Fig. 6**).** This scaffold was embedded with iron-based MOFs (MIL88), which facilitated a Fenton reaction within the acidic TME, generating ROS. The ROS induced oxidative stress, triggering apoptosis in tumor cells.

The CQ@CAS scaffold, loaded with CQ, an autophagy inhibitor, not only exerts direct chemotherapeutic effects but also suppresses autophagic flux, as indicated by increased LC3B accumulation, thereby inhibiting tumor cell survival mechanisms. Autophagy refers to the cellular stress mechanism where cells, upon engulfment of foreign materials or particles, experience injury as a response to expel the inflammatory substance by autophagic digestion. The scaffold also serves as an antigen depot, capturing TAAs released during cell death. These TAAs play a critical role in stimulating DC maturation, promoting T-cell activation, and enhancing immune cell infiltration into the tumor. This antigenic release and ROS generation work in tandem with the scaffold’s antigen capturing capability to amplify ICD, triggering a robust anti-tumor immune response, inhibiting GBM relapse. The proposed scaffold based therapeutic system is feasible and highly appropriate for postoperative GBM therapy for the removal of residual tumor by placing CAS into the resected void/cavity, which would not only carryout Fenton-like reaction for a prolonged time but also facilitate antigen capture and sustained release of antigens for a robust and elongated immune response. The scaffold combines localized drug delivery with immune modulation, offering a promising strategy to reduce tumor recurrence and improve patient prognosis.

This work holds significant promise for post-surgical GBM treatment by combining drug delivery, ROS generation, and immune modulation to improve patient outcomes. Its ability to inhibit autophagy and stimulate anti-tumor immune responses offers a compelling approach. However, challenges such as reproducibility, and precise ROS control must be addressed for clinical translation. Optimizing antigen capture, drug release, and tumor integration will be crucial for maximizing its therapeutic potential. Furthermore, microneedle-aided delivery systems have been developed to enhance immunotherapy by overcoming barriers such as TAMs, which are key players in hindering immune cell infiltration. In response to the challenges posed by GAMs, which prevent DC infiltration and sustain an immunosuppressive M2 phenotype, Zhang et al. [250] constructed an innovative immunomodulatory microneedle-based delivery system. This method aims to improve phagocytosis and prevent postoperative GBM recurrence. The system utilizes self-assembled NPs composed of siRNA, cell-penetrating peptide (CADY), and anti-Siglec antibodies to target oncostatin M (OSM), a factor secreted by GAMs that promotes the M2 macrophage phenotype. These NPs, named GBM-associated macrophage-activating NPs (GMANs), are combined with CpG oligonucleotides and encapsulated in GelMA, forming GMAN@CMNs microneedle arrays designed for targeted delivery. The combination of CpG and ODNs significantly enhanced DC and T-cell infiltration into residual tumor sites, boosting immune responses, and minimizing neurological damage. This dual action counteracts the immunosuppressive TME, reducing GBM recurrence risk after surgery. The microneedle system targets OSM and uses CpG oligonucleotides to strengthen immune responses. OSM targeting in GBM immunotherapy is an evolving approach which has been studied for its role in tumor proliferation and immunomodulatory effects via STAT3 [260]. CpG oligonucleotides have been scrutinized in GBM patients and results suggest modest efficacy. Combining anti-Siglec antibodies and CpG oligonucleotides into microneedles specifically GBM displays promise for GBM therapy [261]. The ability to consistently target GAMs and deliver therapeutics precisely within tumor sites is hindered by the dynamic and heterogeneous nature of TAMs. Additionally, potential side effects from localized toxicity could limit repeated use, requiring careful optimization. While the TME variability across regions presents an added complexity in ensuring uniform effectiveness of the therapeutic strategy. Overcoming these hurdles will be essential for translating this promising approach into clinical practice.

### Biomimicking NPs and biologically derived materials

Biomimetic NPs have proved to be a promising therapeutic model for GBM due to their pathogen/bio-mimicking properties and the presence of biological materials for the targeted activation of innate immunity, promoting immune evasion, and BBB penetration [262–264]. In recent times, researchers have employed surface modification of NPs with cell membranes, which has been a common practice for enhanced circulation and targeted delivery of therapeutic agents [265, 266]. Thus paving a new path for a biocompatible nano-platform for circumventing critical challenges associated with GBM therapy.

Gene editing technologies, including CRISPR-based methods, have also been explored to alter tumor-associated immune cells and suppress tumor-promoting genes, posing a promising avenue for precision medicine in GBM therapy [267, 268]. These technologies allow the targeted modification of immune cells, such as T cells and macrophages, to enhance their anti-tumor activity and overcome immune suppression within the TME. CRISPR-based gene editing system has brought in a revolution by making massive strides in the field of molecular biology by enabling precise and programmable alterations to DNA [269]. Its applications in cancer treatment are up-and-coming, as it offers the potential to rectify oncogenic alterations, interrupt cancer-driving genetic factors, and augment immune response [269]. However, targeted and effective delivery of CRISPR constituents is still a significant hurdle in clinical translation. Biomimetic NPs, which mimic natural biological materials such as cell membranes or extracellular vesicles, may possibly be a powerful tool to improve the delivery and functionality of CRISPR components [270]. These NPs not only exhibit biocompatibility but also evade the immune system and are capable of targeting tumors, offering a synergistic approach to gene editing-based therapies. The combination of CRISPR-Cas with biomimetic nanomaterials holds transformative potential for precision oncology. In tumour therapy, gene editing has been employed to downregulate and upregulate cytokines and proteins, which not only suppress tumours but also significantly enhance innate immune response against cancer[251, 252]. Biomimetic materials, such as cancer cell membranes and EVs, can encapsulate CRISPR-Cas9 ribonucleoproteins and deliver them directly to cancer cells via active targeting, curtailing off-targeting and systemic toxicity [271]. It has also been of tremendous use in screening novel drugs and the resistance mechanism posed by tumours to counteract them [272]. This technique enables circumventing biological hindrances, such as immune clearance and cellular uptake inefficiency.

For instance, NPs surface modified with tumor cell membranes enable homotypic targeting, allowing NPs to accumulate in the tumor specifically [273]. Additionally, immune cell-mimicking NPs can activate or modulate the innate immune system, complementing the gene editing effect [274]. This allows for gene correction and immune modulation in parallel. Moreover, CRISPR-containing biomimetic NPs can be designed for stimuli-responsive release, enabling gene editing to take place specifically within tumor sites [275]. Such precision eliminates the chances of genomic alterations in healthy tissues and enhances therapeutic safety. CRISPR-based gene editing, facilitated by stimuli-responsive biomimetic NPs, represents a novel approach in cancer therapy [276]. By editing the genome of tumor cells or immune cells, CRISPR-based strategies can enable the development of personalized treatments that target the unique molecular features of a patient’s tumor, potentially improving therapeutic outcomes and reducing resistance [277, 278].

**Fig. 7:**
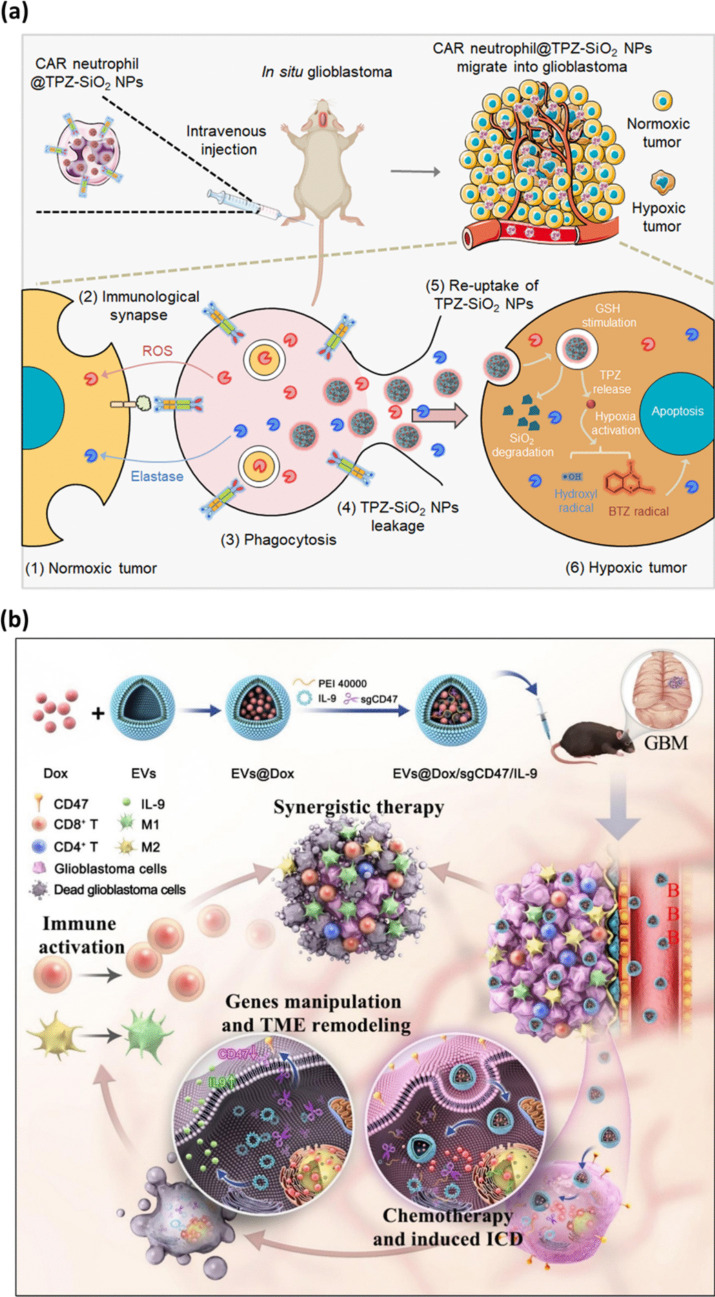
(**a**) Illustration of CAR-neutrophil@R-SiO2-TPZ NPs targeting external normoxic tumor cells. The CAR-neutrophils form immunological synapses with the tumor cells and kill them through phagocytosis. CAR-neutrophils release R-SiO2-TPZ NPs into the hypoxic tumor microenvironment, effectively killing the tumor cells [251]. **b** Illustrates the construction and therapeutic mechanism of the EVs@Dox/sgCD47/IL-9. The EVs cross the BBB and specifically target GBM cells to facilitate chemotherapy and simultaneously gene editing via CRISPR/Cas9 to reduce CD47 expression and increase IL-9 levels, reshaping the TME to induce ICD [252]

Among the most promising approaches, EV-based delivery systems have confirmed significant potential in improving drug transport across the BBB. EVs are naturally occurring nanocarriers arising from lipid bilayer cell membrane capable of encapsulating chemotherapeutics, genetic materials, and immunomodulatory agents [279, 280]. Their innate capability to cross the BBB efficiently, combined with their biocompatibility and low immunogenicity, marks them as ideal candidates for the treatment of GBM. EV-based systems have the potential to enhance drug delivery directly to the tumor site, thereby increasing therapeutic efficacy and reducing side effects [281]. Another approach, the incorporation of engineered immune cells, such as CAR-modified neutrophils, denotes a novel frontier in GBM immunotherapy. The CAR construct comprised of GBM-targeting domain, CLTX, which was obtained by genetically engineered human pluripotent stem cells (hPSCs) into via CRISPR/Cas9-assisted repair at the AAVS1 locus, ensuring sustained receptor expression and formation of CAR-neutrophil. Building upon this concept of targeted immune modulation, Chang et al. [251] developed a CAR-neutrophil-based targeting and drug delivery system for brain tumor therapy **(**Fig. 7a**)**. The CAR construct comprising GBM-targeting domain, CLTX, which was obtained by genetically engineered human pluripotent stem cells (hPSCs) into via CRISPR/Cas9-assisted repair at the AAVS1 locus, ensuring sustained receptor expression and formation of CAR-neutrophil.

The generation of CAR-neutrophils from human pluripotent stem cells was successfully confirmed through RT-PCR and flow cytometry, demonstrating the expression of CLTX-IgG4 on the neutrophil surface. Furthermore, the system integrated SiO_2_ NPs loaded with tirapazamine (TPZ), a hypoxia-activated prodrug. TPZ is activated in the hypoxic regions of GBM, enhancing the therapeutic effect by selectively targeting tumor cells in these areas. By combining CAR-modified neutrophils, which provide tumor-specific targeting, with hypoxia-activated drug delivery, the platform not only directly targets GBM cells but also amplifies therapeutic efficacy in the hypoxic TME. While this approach holds great promise for GBM and potentially other deadly diseases, the challenge of overcoming the BBB remains a major hurdle for its clinical application. This work presents a novel and potentially transformative approach for GBM, the hurdles outlined above are not insignificant. The scalability of generating CAR-neutrophils from human pluripotent stem cells remains a key challenge for broader clinical application, and addressing the variability in hypoxic tumor regions could be critical for ensuring consistent drug activation. Additionally, overcoming the BBB remains one of the most persistent challenges in GBM treatment, and further advancements in targeting strategies or BBB penetration methods will be crucial to making this therapy clinically viable.

Immune-based approaches have gained significant attention in recent years as a way to enhance GBM treatment. Engineered immune cells, such as chimeric antigen receptor T cells (CAR-T cells) and macrophage-targeting dendrimer-based methods, have been aimed to overcome the immunosuppressive nature of the TME and enhance anti-tumor activity [282, 283]. CAR-T cells, which are engineered to target TAAs, have shown potential in treating hematological malignancies and are now being adapted for use in solid tumors like GBM [284]. Additionally, dendrimer-based methods can be engineered to specifically target tumor-associated macrophages, which are key players in the GBM immune evasion process [285]. In a notable study an EV-based platform demonstrated in **(**Fig. 7b**)** is the EVs@dox/sgCD47/IL-9 system, where Yang et al. [252] and team employed GBM-derived EVs to breach the BBB. The GBM-derived EVs were integrated with multiple systems, including encapsulation of DOX, CRISPR-Cas9-mediated gene editing targeting CD47, and an interleukin-9 (IL-9) overexpression vector, to modulate the immune response. DOX induces ICD, activating APCs and triggering an immune response against the tumor. The CRISPR-Cas9 system reduces CD47 expression, making GBM cells more susceptible to immune attack by enhancing macrophage-mediated phagocytosis. Furthermore, IL-9 enhances the recruitment and activation of cytotoxic T lymphocytes (CTLs) and natural killer (NK) cells, promoting an immune-activated TME.

Though preliminary studies performed on mice reveal promising results, the transition of CRISPR-based technologies into real time clinical use face multiple critical challenges. Targeted delivery into immune-privileged organs is one of the major obstacle which drastically reduces the therapeutic efficiency [286]. Off-targeting is another concern which would lead to mutations there by bringing a need for more strong regulatory policies and improving targeting specificity [287]. Moreover, Cas9 protein and viral vectors may induce immunogenicity thereby leading to adverse immune reactions and reducing long-term therapeutic efficacy [288]. By addressing delivery challenges and enhancing specificity, this strategy paves the way for more effective, personalized, and less toxic treatments, with ongoing research pushing the boundaries of what is possible in gene-based cancer interventions.

Preclinical works revealed the successful penetration of the BBB**,** enabling efficient delivery of the therapeutic agent to the tumor site. The downregulation of CD47 aided immune cell infiltration, while IL-9-mediated immune stimulation further amplified tumor clearance. Moreover, this EV-based system demonstrated excellent biocompatibility, prolonged circulation time, and minimal toxicity due to its ability to evade rapid clearance by the mononuclear phagocyte system. Unlike synthetic NPs, EVs provided superior stability and lower immunogenicity, improving overall therapeutic efficacy. Furthermore, the CRISPR-Cas9 system was optimized to minimize side effects, ensuring a safer gene-editing method for cancer therapy. The EVs@dox/sgCD47/IL-9 system combines chemotherapy, CRISPR-Cas9 gene editing, and immune modulation to enhance immune-mediated tumor clearance in GBM. While this method effectively penetrates the BBB and promotes APCs activation, challenges in EV engineering, cargo loading efficiency, and large-scale production remain barriers to clinical translation. This work represents a promising nanoplatform that combines chemotherapy, immune modulation, and gene editing for enhanced GBM treatment. This strategy successfully overcomes multiple barriers in GBM therapy, including poor BBB penetration and tumor immune evasion. Nevertheless, further optimization is needed to improve EV engineering and increase clinical feasibility, particularly in terms of mass production, precise targeting, and safety concerns related to CRISPR-Cas9 gene editing. Future studies integrating this platform with checkpoint blockade therapies could further enhance its therapeutic potential.

Another significant innovation in overcoming brain targeting challenges is the advancement of ligand-functionalized NPs and cell-penetrating peptide-based delivery systems. These methods focus on enhancing BBB penetration and selective tumor targeting. By attaching ligands or peptides that specifically recognize receptors overexpressed on GBM cells, these NPs can dynamically target tumor cells, improving drug delivery precision [289, 290]. This method not only enhances therapeutic outcomes but also reduces the unintended effects on healthy cells [291]. Spiky gold NPs, acting as artificial antigen-presenting cells (aAPCs), have shown potential in successful T-cell activation within the tumor. The unique structure of these NPs offers a larger surface area for ligand presentation, letting for more effective T-cell priming and activation [292, 293]. This technique enhances the immune response against GBM by ensuring robust T-cell activation, eventually improving the efficacy of immunotherapies and potentially leading to better patient outcomes [294]. Cheng et al. and team developed gold NPs that were camouflaged with rabies virus glycoprotein and named them RVG@GY [253]. The function of the gold NPs was to deliver palbocilib, disrupt tumor cell junctions to enable T-cell infiltration, and facilitate deeper penetration of the nanostructure upon exposure magnetic field.

### Stimuli responsive systems

Stimuli-responsive systems, such as pH-sensitive, thermosensitive and ultrasound activable carriers, influence the unique characteristics of the TME to enable controlled drug release and disrupt BBB [295–298]. The acidic nature of the TME within the tumor can trigger the release of encapsulated agents, improving the effectiveness of the treatment [299]. Moreover, externally irradiated energies such as NIR, magnetic field, and ultrasound-mediated extrinsic stimuli responsive nanomaterials have been studied widely as GBM interventions and for BBB disruption by means of ROS generation. Both NIR and magnetic field responsive NPs induce cell death by hyperthermia and ROS while ultrasound responsive generate ROS and are capable of disrupting BBB and cancer cell membrane [300–302]. External magnetic field not only triggers hyperthermia but can also be used for targeted delivery of NPs to tumor site [303]. These systems offer a promising approach to achieve targeted, on-demand drug delivery in GBM therapy, improving both drug bioavailability and therapeutic outcomes [304].

**Fig. 8:**
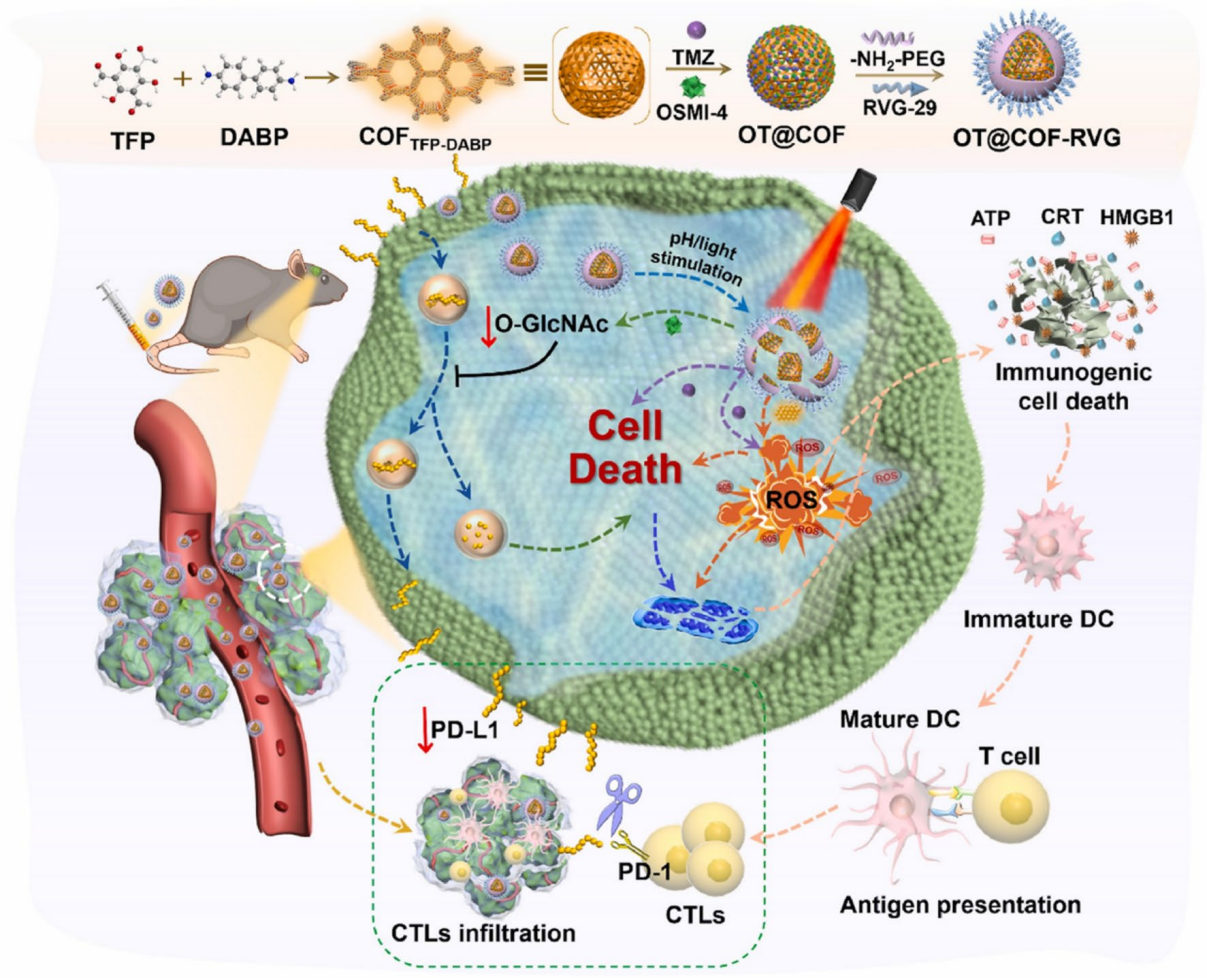
Depiction of OT@COF-RVG synthesis and mechanism. The hybrid system combines organic frameworks and RVG peptides to facilitate targeted delivery to GBM cells. The organic framework mediates immune checkpoint blockade and photodynamic immunotherapy, inhibiting tumor progression and enhancing immune responses against GBM cells [254]

COFs have been explored as new, light-activated platforms for the treatment of GBM, their ability to generate ROS under NIR irradiation. The generation of ROS induces ICD, which not only directly kills cancer cells but also releases TAAs. These antigens can boost immune responses, making a dual impact: both cytotoxic and immunomodulatory. Yan et al. [254] advanced this concept by introducing the OT@COF-RVG platform **(**Fig. 8**)**, specifically constructed to enhance GBM treatment. This platform integrates a pH-sensitive excited-state intramolecular proton transfer (ESIPT) mechanism, generating ROS when activated by light. The OT@COF-RVG nanoplatform combines temozolomide (TMZ), O-GlcNAc transferase inhibitor (OSMI-4), and rabies virus glycoprotein (RVG-29) to address key challenges in GBM therapy, including BBB penetration and immune suppression. RVG-29 empowers BBB penetration via binding to the α7 subunit of nicotinic acetylcholine receptors which are abundantly expressed on glial cells, brain endothelial cells, and neurons, thus facilitating targeted delivery to brain and reducing the chances of off-targeting [305]. OSMI-4 moderates immune checkpoints to enhance immune responses, complementing TMZ's cytotoxic effects. Furthermore, the platform includes PEG for stability and prolonged circulation. This dual strategy induces tumor cell death via ROS-mediated ICD and promotes T-cell activation, stimulating anti-tumor immunity. Preclinical studies display improved survival rates, minimal toxicity, and specific targeting of GBM cells. This work's key challenges include controlling ROS generation to avoid healthy cell damage and ensuring consistent BBB penetration across patients. While TMZ and immune checkpoint modulation enhance efficacy, further integration of therapeutic agents and assessment of long-term stability are needed for clinical success.

Yalamandala et al. [255] advanced their research into nanomedicine by developing a NIR-II activatable membrane-disrupting nanosystem constructed to amplify immune responses in GBM. Their system employed copper monosulfide (CuS) nanoballs (CuS NBs), composed of CuS nanoflakes embedded within a poly (ethylene glycol)-benzoic imine-octadecane (mPEG-b-C18) polymer matrix. In this study CuS NBs were delivered into the cranial cavity to the tumor via CED, where the pressure employed augments CuS NBs distribution across the BBB and deep tumor tissues. The use of CED facilitated delivery of CuS NBs specifically to the tumor as CuS NBs were engineered to undergo charge conversion in the acidic TME. This conversion enhanced NPs permeability, due to the membrane disrupting capability of mPEG-b-C18 coated on CuS allowing for more efficient tumor penetration and targeting. The system's key feature is nanoparticle-induced endothelial leakage (NanoEL), which enhances CuS NBs accumulation in GBM by disrupting the tumor’s dense vasculature. NIR-II irradiation induces hyperthermia, triggering apoptotic cell death and releasing tumor TAAs, which are captured by the amine-functionalized polymeric coating of CuS NBs. This sustained antigen capture promotes long-term immune activation, recruiting DCs and T cells into the tumor. This work offers promising tumor-targeting hyperthermia and immune activation for GBM treatment. While the NanoEL mechanism enhances NPs accumulation. The heterogeneous TME complicates the efficient delivery of NPs, and optimizing NIR-II irradiation is necessary to minimize damage to healthy tissues. Overcoming these issues is essential for advancing this strategy into clinical practice.

Focused ultrasound combined with microbubbles has developed as a non-invasive strategy to transiently disrupt the BBB, allowing enhanced drug penetration into the tumor. By using focused ultrasound to construct temporary pores in the BBB, this method enables the delivery of therapeutic NPs directly to the brain, improving the accessibility of drugs that the BBB would otherwise restrict [306]. This method is particularly valuable in the context of GBM, where ensuring that therapeutics reach the tumor site is one of the biggest barriers to effective treatment [307]. Montorsi et al. [256] formulated an ultrasound responsive piezoelectric nanomedicine surface modified with GBM cell membrane for active targeting and electrical stimulation of microglia cells and named it CM-PNPs. The proposed CM-PNPs possessed camouflaging capabilities to specifically recognize GBM by means of homotypic targeting. The study demonstrated effective stimulation of glial cells and their polarization into the M1 phenotype via electrical impulse. The approach involves wireless modulation of TME for immunotherapy by M1 microglia, affirmed by a dramatic increase in IL-6, IL-12, and nitric oxide synthase (iNOS) along with upregulation of TNF-α and IFN-γ, resulting in a pro-inflammatory response [308–310]. Other APCs such as CD86, CD80, and CD40 were quantified to confirm maturation of DCs, which promoted the activation and infiltration of cytotoxic T-cells, enhancing anti-tumor activity [311].

## Hepatocellular Carcinoma (HCC)

The hepatic system plays a vital role in maintaining homeostasis by metabolizing glucose, lipids, and proteins. Thereby aiding in detoxification, maintaining the equilibrium of energy, production of essential biomolecules, and acting as a micronutrient repository [312, 313]. Hepatocellular carcinoma (HCC) presents a major challenge in diagnosis as it occurs simultaneously with existing hepatic disorders such as metabolic dysfunction, hepatitis B and C infection, non-alcoholic fatty liver, and cirrhosis [314–316]. The occurrence of HCC together with other hepatic disorders makes it problematic to concurrently treat multiple disorders with limited intervention alternatives. Apart from that, HCC poses the risk of drug resistance and is vulnerable to recurrence post-surgery and chemotherapy [317, 318]. Moreover, the liver itself influences the drugs by detoxification and metabolism thus hindering therapeutic efficacy. The first TKI, sorafenib, approved by the FDA for HCC therapy has shown limited efficiency with rapid resistance in multiple HCC patients [319–321]. Likewise, individuals treated with TKIs such as cabozantinib and lenvatinib have benefited minimally, with a low survival rate [322, 323]. ICI nivolumab has shown limited benefits upon its application as second-line therapy, thereby urging researchers to urgently need HCC medication [324]. Moreover, the liver is prone to metastasis arising from the gastrointestinal (GI) tract, posing a major challenge [325, 326].

### Characteristics of HCC

HCC, being a highly malignant form of cancer with the tendency to metastasize, it is vital to understand the interactions within TME. The primary components that result in a poor prognosis in HCC are cancer-associated fibroblasts (CAFs), hepatic stellate cells, cancer stem cells, and TAMs [327]. Cytokines such as TGF-β and IL-6 that facilitate suppression of the immune system and promote epithelial-mesenchymal transition (EMT) are secreted by CAFs [328]. Thereby promoting migratory and invasive properties. M2 phenotype TAMs produce angiogenic factors such as VEGF-A, resulting in neovascularization and proliferation of HCC [329]. HCC metastasis involves various pathways such as JAK/STAT, Wnt/β-catenin, TGF-β/Smad, and PI3K/Akt/mTOR, which play roles in proliferation, immune evasion, and EMT [330]. The Wnt/β-catenin signaling facilitates EMT by increasing and decreasing vimentin/N-cadherin and E-cadherin, respectively [331]. Mutations in lipid metabolism further aids in meeting energy demand of metastatic HCC increasing invasiveness [332].

Pathological understanding is necessary for delivering therapeutic agents via selective targeting for efficient uptake by HCC. To facilitate the same, researchers have been extensively working on multiple strategies, which include the exploitation of surface receptors and EPR effect for effective uptake by HCC [333]. Furthermore, understanding the microenvironment of HCC in the past decade has been vital for actuated advancements in immunotherapy.

### Divergence in vascular normality

Macromolecules and NPs exhibit a greater permeability and retention in tumors via the EPR effect, with HCC posing no exemption [334]. The leaky vasculature of the tumor promotes uptake due to loosely packed endothelial cells with greater intercellular spaces [335, 336]. The EPR effect is a passive targeting technique that is being exploited by researchers for the effective accumulation of NPs which depends upon various factors such as surface charge, biocompatibility, size, and shape of NPs [337, 338].

Smaller NPs exhibit prolonged circulation by evading mononuclear phagocyte system (MPS) mediated clearance. MPS are a part of immune system primarily comprising of blood monocytes, bone marrow progenitors, and tissue macrophages, which possess the capability to identify danger signals [339]. Apart from that they diffuse at a greater rate displaying more prominent accumulation into the tumor [340]. NPs with sizes smaller than 8 nm get cleared by the renal system [341]. The cell membrane possesses a negative charge thereby hindering penetration of NPs with low zeta potential [342, 343]. The lower surface charge of NPs increases the risk of hydrophobic collapse leading to an increase in the size of the nanoscaled aggregates thus hindering the uptake of NPs. The collapsing of NPs could be averted by the spatial repulsion of ligands on the surface of NPs [344]. Over year’s multiple studies have demonstrated that nanorods exhibit greater penetration than nanospheres equivalent to 1.7 times. Nanostructure with rod and cages shape penetrates the tumor core efficiently when compared with sphere and disk-shaped NPs [345]. Underlying factors such as haemoglobin oxygenase, cyclooxygenase, bradykinin, matrix metalloproteinases, nitric oxide peroxynitrite, TNF-α, vascular epidermal growth factors, and carbon monoxide influence the EPR effect [346]. In recent years’ therapeutic agents and NPs have been delivered by wrapping the same with cell membranes including that of platelets, cancer cells, and red blood cells (RBCs) to escape phagocytosis and enhance EPR [347–350]. At the time studies have also reported active targeting of nanoagents for HCC. This includes designing of NPs with the ability to target tumor vascular endothelium as they express a certain surface marker that can act as ligands for targeting. VEGF has been exploited in order to target HCC and ameliorate NPs tumor penetration and therapeutics [351].

### Upregulated glycolytic activity

The liver plays a vital role in modulating of glucose levels throughout the body. Glucose is stocked in the form of glycogen upon abundant availability and discharges glucose into the blood upon scarcity thereby regulating glucose in the circulatory system. The liver releases glucose by facilitating glycogen catabolism during fasting and at times of high energy requirements [352]. Even under the availability of oxygen, HCC depends on glycolysis for the production of energy a popular event known as the Warburg effect [353]. This deviation from the oxidative phosphorylation to glycolysis in an oxygen-surplus environment permits HCC to generate energy efficiently. HCC expresses various glycolytic genes such as hexokinase (HK), glucose transporters, (GLUTs), and pyruvate kinase (PK) which aid in glucose anabolism, catabolism, and uptake. The glucose uptake into the cytoplasm is facilitated by GLUT1-4 transporters through the plasma membrane [354]. After reaching the cytoplasm of the cell the glucose is transformed into glucose-6 phosphate (G6P) [355]. HKs are actively involved in the process of conversion due to upregulated HK2 in HCC which is associated with further prognosis of cancer [356]. The capability of HK2 to augment glycolysis is due to its capability to bind with voltage-dependent anion-selective channel protein-1 situated in mitochondria thereby increasing its access to adenosine triphosphate (ATP) [357, 358]. The exhaustion of HK2 impedes tumor growth and surges cell death [312, 314]. The enzyme PK transports phosphate to adenosine diphosphate (ADP) to obtain ATP. HCC formation is related to the enhanced presence of M2 isoform of PK [359, 360]. Lactate dehydrogenase (LDH) assists the transformation of pyruvate to lactate leading to high levels of LDH in blood which is linked to poor prognosis in individuals suffering from HCC [361, 362]. Elevated levels of lactate and LDH are the key characteristics of HCC. Studies suggest that reduction in metabolites such as glycerol-3 and 2-phosphate, malate, and glucose, along with simultaneous enhancement in the glycolysis [363]. Both Wnt/β-catenin and PI3K/Akt/mTOR pathways amplify glycolytic enzymes in HCC [319, 364, 365].

### Markers of HCC

Surface markers are not only essential for diagnosis but also can be exploited for targeting tumors. The HCC cells present glypican-3 (GPC3) on the surface which aids in understanding the prognosis of HCC in patients. Chemokine receptor 4 (CXCR4) plays an essential role in tumor cells as it aids them in escaping the immune system and promotes proliferation and angiogenesis. Poor prognosis is observed in HCC patients with overexpression of CXCR4 [366]. The surface proteins such as platelet-derived growth factor receptor β (PDGFRβ) and insulin-like growth factor receptor II (IGFRII) are amplified by CAFs. Studies have shown that NPs coated/surface-modified with mannose 6-phosphate or peptides can specifically target and accumulate in HCC which significantly improved the therapeutic outcome [367].

### Nanoparticles in HCC immunotherapy

With HCC being the cancer that is usually diagnosed at advanced stages is conventionally treated by surgical resection/liver transplant followed by chemotherapy and radiotherapy. But the same is restricted by donor availability, tumor volume to be resected for effective regrowth and functioning of the liver [327]. Though TKIs have been gaining interest TKIs alone provide minimal benefits and pose the threat of systemic side effects [368, 369]. Adoptive cell therapy, cytokine therapy, immune checkpoint therapy, vaccines, and oncolytic viruses are various forms of immunotherapy used to treat HCC. NPs can be tailored as per requirement and can be utilized for the destruction of blood vessels and bringing normalcy in cancer cells via the transport of CRISPR into the nucleus for editing genes. NPs can also be utilized to activate innate immunity, aid nucleic acid therapy, and boost adjuvant therapy that targets the hepatic system. Recent advancements in liver cancer immunotherapy have led to experimentation with innovative techniques as shown in Table 3.

**Table 3:** Nanomedicines for liver cancer immunotherapy

Particles	Material	Cell Line/Tumor Model	External Energy Source	Administration Route	Description
MPFD	ZnCoFe_2_O_4_@ ZnMnFe_2_O_4_	Hepa 1–6 cells	Alternating magnetic field	Tail vein	Mild hyperthermia activates HSP70 in cancer cells, stimulating STAT5 and JAK3 signalling pathway activating NK cells [370]
OCA-NPs and 5β-CA-NPs	PMBEOx-NH_2_	H22 cells	None	Tail vein	Delivery of obeticholic acid and 5β-cholanic acid 3 for robust activation T-cell and NK-cell [371]
AuNCs	Gold NPs loaded with Ansamitocin P3	Hep55.1c cells	NIR-I	No in vivo assay performed	PTT, anti-PDL1, and chemotherapy synergistic in vitro assessment [372]
GH-PID	Aldehyde hyaluronan, hydroxyethyl chitosan, and galactosamine-hyaluronan	HepG2 and Hepa1-6 cells	NIR-I	Tail vein	The donut-like nanoparticle induced ICD via photo-chemotherapy and PD-1 blockade [373]
CA4-NPs	PLG-g-mPEG encapsulating combretastatin	H22 subcutaneous tumor models	None	Tail vein	CA4-NPs in combination with a VEGF inhibitor (DC101) for liver cancer suppression and activation of CD8^+^ cells [374]
R848@M2pep-MPs_AFP_	Macrophage cell microparticles	Hepa1–6 and H22 cells	None	Intravenously injected	The nanoplatform reprogrammed TAMs into an M1 phenotype, and combined with a TLR agonist, to promote ICD [375]
807-NPs	1,2-dioleoyl-sn-glycero-3-phosphocholine (DOPC)	HCA-1 cells	None	Intravenously injected	CXCR4 antagonist inhibits Akt and mTOR, repolarizing macrophages to M1 phenotype, activating CD 8 cells [376]
AIE-Mito-TPP NP	Mal-PEG-PLGA	Hepa1–6 and H22 cells	None	Tail vein	Mitochondrial membrane damage mediated activation of cGAS-STING promoting DCs maturation and M1 polarization [377]

### Activation of NK cells

The acidic microenvironment of tumor and neutrophil extracellular traps (NETs) reduce the efficiency of NK cell infusion in postoperative cancer treatment. This could be overcome with neutralizers and hydrogels containing NETs lyase to boost NK cell infusion to inhibit the recurrence of HCC post-surgery. The study reports the fabrication of injectable hydrogels at the region of the resected cavity to form a mucoadhesive gel which clogged bleeding. The hydrogel also neutralized the acidic TME as a result releasing DNase I and diminishing tumor infiltration by immunosuppressive cells to deteriorate NET. This augmented the NK cell's activity to inhibit tumor recurrence without posing cytotoxicity towards healthy cells [378].

Determination of NK cell viability is crucial in gauging therapeutic efficiency and quantifying the same researchers developed an imaging strategy by scaling NIR-II fluorescence imaging for real-time tracking and viewing of pericyte viability. Nanoprobes consisting of lanthanide-based material were covered with IR 786 s and ROS responsive dye, which could labele NK cells. The signal of NIR-II fluorescent dye was directly proportional to the concentration of ROS, exhibiting a correlation between NK cell viability, allowing for real-time quantification of cell viability for effective NK cell mediated immunotherapy [379]. In an attempt to activate NK cells, Jiang et al. [370] reported a magnetic field-responsive NPs with the capability to induce NK cell activation, infiltration, and proliferation upon irradiation of AMF facilitated by IL-2 upregulation. They employed ZnCoFe_2_O_4_@ZnMnFe_2_O_4_ nanoparticles (MNPs) and surface-modified it with polyethyleneimine (PEI) and FA. The NPs were further grafted with plasmid DNA (pDNA, HSP70-IL-2-EGFP) to obtain MNPs@PEI-FA/pDNA and termed the therapeutic system as MPFD. The doping of Zn2^+^ facilitated a controllable magnetothermal effect by means of exchange-coupled magnetism. The surface modification of MNPs with PEI-enabled pDNA loading via an electrostatic interaction between positively and negatively charged PEI and nucleic acid, respectively. FA, being a popular agent for cancer targeting in drug delivery systems, was exploited for the same [380, 381]. The presence of FA enables MPFD to precisely targets liver cancer and minimizes off-targeting for localized immune activation unlike IL-2 causing systemic toxicity. The proton sponge effect posed by PEI set off the intranuclear transport of pDNA upon lysosomal escape of MPFD [382, 383]. Irradiation of AMF generated mild hyperthermia in orthotropic liver cancer models to trigger HSP70 and secreted IL-2, thereby expanding and stimulating NK cells via JNKs/c-Jun and IL-2R/JAK1/JAK3/STAT5 signaling cascade correspondingly. The copious discharge of effector molecules such as perforin (PRF1) and granzyme B (GzmB) improve NK cells toxicity towards tumor cells by amplification of NKG2D receptor leading to drastic reduction in tumor volume. Importantly mild hyperthermia elevated retinoic acid early inducible (Rae-1) proteins, aiding in tumor recognition by NK cells. Moreover, studies suggest that integration of NPs with antibody, stimulating DCs, IL-2, and interleukin-5 (IL-5), and can trigger the activation of NK cells [384, 385]. Apart from the use of antibody for the activation of immune response, in recent years semi-synthetic steroids have gained interest for receptor modulation to provoke anti-tumor immune response by balancing bile acids in case of HCC.

A dysregulation in bile acids involving a decrease in primary and simultaneous increase in secondary bile acids is a mechanistic association with hepatocellular carcinoma. Ji et al. [371] and co-workers theorized controlling the primary and secondary bile acid receptors in the hepatic system is an unconventional yet accurate technique to circumvent microbiota for regulating the bile acid signalling of hepatocellular carcinoma. Farnesoid X receptor (FXR) acts as a receptor for primary bile acids and G-protein-coupled bile acid receptor 1 (GPBAR1) for secondary bile acids which are profoundly found in the gastrointestinal (GI) tract thereby reducing chances of off targeting [386–388]. They employed polyoxazole NPs for delivering agonist obeticholic acid (OCA) and antagonist 5β-cholanic acid 3 (5β-CA) to FXR and GPBAR1, respectively [389]. This restores the production of primary bile acids and reduces secondary bile acids. Activation and inhibition of FXR GPBAR1 stimulates the upregulation of chemokines CXCL16/9/10, resulting in expression, expansion, and infiltration of NK, natural killer T (NKT), and T-cells arousing a robust and efficient immunotherapy in HCC bearing mice.

### Nanoparticles as immune checkpoint inhibitor

NPs boost the activity of immune ICIs and significantly decrease off-targeting. HCC tumors treated with iron oxide NPs and anti-PD1 complex reduced the immunosuppressive microenvironment leading to tumor suppression via synergistic therapy [390]. The formulation not only successfully targeted the tumor but also activated T-cells by means of polarization of macrophages to M1. Orthotropic mice models revealed enhanced immunotherapeutic efficiency. Ansamitocin (AP3) is a water-insoluble molecule isolated from Nocardia with capabilities to aid in the production of pro-inflammatory cytokine, T-cell activation, and DC maturation [391, 392]. PTT has been vastly employed for heating of tumors to boost ICDs without harming surrounding healthy cells. In a study Cheng et al. reported PTT responsive NPs combined with AP3 to enhance immune response. The study revealed enhancement of mature DCs thereby improving therapeutics [372].

ICI based immunotherapy have exhibited satisfactory results only in a few individuals with tumors exhibiting characteristics such as enhanced T-cell infiltration there by failing in individuals with cold tumors. Thus, a trend has been developed over the years to reverse the cold tumor into a hot one to induce ICD in tumor cells. Researchers developed a multifunctional nano-platform consisting of galactosamine/hyaluronan incombination with indocyanine green (ICG) co-loaded with DOX with PTT, chemotherapeutic, and PDT capability to augment immune response in HCC. The donut shape NPs were termed as GH-PID and upon the combination with α PD-1-based immunotherapy the cancer intervention inhibited infiltration and proliferation of MDSCs and augmented DC maturation [373].

Anti-PD-1 therapy is enhanced significantly upon application of vascular endothelial growth factor receptor (VEGFR 2) inhibitor DC101. An improvement in CD8^+^ infiltration was observed at tumor site and NPs fabricated using poly (L-glutamic acid)- graft-methoxy poly (ethylene glycol)/combutane A4 exhibited capabilities to modulate imbalance between T-cells and tumor load. A remarkable tumor suppression was observed in groups treated with CA4-NPs + DC101 in combination with PD-1 antobody when compared with anti-PD-1 alone thus suggesting the occurrence of vascular disruption at tumor site and normalcy-boosted anti-PD-1 therapy [374].

### Alternative strategies for the induction of immunogenic cell death (ICD)

Convectional NP based therapeutic strategies focus on posing a cytotoxic effect via drug and antigen delivery. In the recent years improvements involve alternative approaches, where researchers have tried to exploit TME and modulate innate immunity to promote ICD. The tactics employed include blocking of pathways, TME remodeling, and targeted delivery of therapeutic agents. This section discusses the innovative techniques and unconventional therapeutic NPs that effectively promote ICD via activation of innate immune response.

In the study, Zhang et al. [375] and team fabricated microparticles (MPs) using macrophages overexpressing alpha-fetoprotein (AFP) which were further altered with peptide (M2pep) to target M2 macrophage [393, 394] rather than leukocytes. The MPs were further loaded with a TLR7/8 agonist resiquimod (R848) for cancer immunotherapy and termed the HCC intervention R848@M2pep-MPs_AFP_. R848 has gained interest of researchers due to its immunomodulatory effect with capability to act as antiviral and anticancer agent [201, 395]. The primary motive of the research was to reprogram TAMs into M1-like macrophages to mitigate the immunosuppressive microenvironment of HCC. Apart from reprogramming of M2-like TAMs, R848@M2pep-MPs_AFP_ acted as an immunopotentiator by presenting AFP antigen to CD8^+^ T cells, consequently leading to stimulation and proliferation of CD8^+^ T cells at the tumor site. The reprogramming of TAMs preserved the intra-tumoral niche, aiding in the retention and differentiation of stem-like CD8^+^ T cells. The consolidation of these properties enhanced anti-PD-1 therapy in HCC.

Building upon this immunomodulatory approach, major strides have been made to reprogram TME and promote antitumor immunity. In recent years, researchers have extensively worked on the development of NPs to target CXCL12/CXCR4 in combination with various cancer therapeutics and have demonstrated antitumor effects along with inhibition of metastasis [396–399]. Cheng et al. [376] designed 807-NPs a lipid-based nano-delivery system to deliver BPRCX807 a CXC motif chemokine receptor type 4 (CXCR4) antagonist. The nano-delivery system was developed with a tannic acid (TA) core which was specifically selected due to its negative charge arising from hydroxyl and galloyl groups along with aromatic rings. The presence of these functional groups and moieties provides TA an effective molecular interaction with cationic compounds such as BPRCX807. The outer coating of lipid (DSPE-PEG) not only enhanced the *in-vivo* structural integrity of 807-NPs, but also ameliorated neoplasm targeting, disposition, and metabolism of BPRCX807. They demonstrated the effective delivery of BPRCX807 into orthotropic HCC-bearing mice models, which blocked the CXCR4 pathway as a result inhibiting activation Akt followed and mTOR pathway. This facilitated the repolarization of M2 phenotype macrophages into M1. The reprogramming of TAMs stimulated robust infiltration of cytotoxic T-cells at the tumor site thereby suppressing both primary and a distal metastatic tumor in HCC-bearing mice.

Advancing beyond chemokine axis inhibition and macrophage repolarization, studies have also revealed tactics targeting mitochondria to trigger immune response via organelle specific stress. Song et al. [377] and team designed a triphenylphosphine (TPP) based therapeutic system with AIE-Mito-TPP as the primary agent for mitochondrial targeting and as a mitophagy agent. They employed poly lactic-co-glycolic acid (PLGA)-PEG-maleimide(mal) nanodelivery system surface modified with matrix metalloproteinase 2/9 (MMP-2/9) responsive PD-L1 peptide as a carrier of AIE-Mito-TPP and named the HCC intervention ^D^PPA-1 M@AIE-Mito-TPP. This is the first report to demonstrate the induction of incomplete mitophagy for the activation of innate immune response in HCC. They exploited the strategy of incomplete mitophagy to facilitate the prolonged release of DNA from mitochondria which activated innate immune response by triggering cGAS-STING followed by DCs activation and reprograming of TAMs.

## Complications associated with nanomedicine-based therapy

Though nano-platform mediated immunotherapy for the suppression of GBM, lung cancer, and HCC has delivered promising results, there is a severe prevalence of various limitations that hinder efficacious therapy. The hindrances arise from the interaction between biological components, manufacturing, and scalability, as well as complications associated with regulatory affairs[400–403].

### Regulatory affairs

The development of regulatory standards for nanomedicine is complicated and is still evolving [404]. Both the FDA and the European Medicines Agency (EMA), are rigorously working on setting up suitable parameters for gauging the safety and efficiency of nanomaterials-based therapeutic systems [405]. But scrutinizing the properties of nanomedicines is quite a challenge under traditional regulatory standards. The primary problem is the lack of well-regulated characterization techniques for nanomedicine under various regulatory jurisdictions[406]. This leads to disagreements not only on the classification of NPs but also on their evaluation criteria [407]. Though serious efforts are being made by the regulatory bodies to bring a uniform and standardized set of regulations, it is still a momentous task to well define and harmonize a refined set of rules. Prioritizing collaboration with international regulatory agencies is the key to addressing challenges associated with standardizing unique characterization techniques for nanomedicines [408]. The other challenge is to establish suitable preclinical procedures for the preliminary evaluation of nanomedicine’s therapeutic mechanisms and efficacy[409, 410]. Conventional toxicological evaluation assays may not completely reveal the distinct biological interactions of nanomedicines, thereby raising demands for specialized assays to assess the determination of long-term toxic effects and a comprehensive study for the biodistribution of nanomedicines [411, 412]. Apart from that, the incorporation of devices and drugs into nanostructures also raises new challenges for the establishment of standards by regulatory authorities, making the nanomedicine-based product be evaluated under a number of regulatory categories [405]. Thereby, demanding a coordinated evaluation by the multiple boards under the agency [408].

Despite these complexities, substantial progress has been made to bring clearer and more transparent standardization procedures for the evaluation of nanomedicines. Both the Expert Group and the Nanotechnology Task Force of the EMA and FDA, respectively, are constantly functioning on the development of a well-defined structure and standard set of rules for the rapid and safe deployment of nanomedicine into the market [408, 413].

### Manufacturing difficulties and large-scale production

The successful transition of nanomedicine from synthesis to real-time application is impeded by the complexities associated with manufacturing and large-scale production. Many nanomedicines that have been successfully synthesized and studied in the laboratories of research centres and academic institutions are very difficult to reproduce under an industrial setup for large-scale production and commercial use [414, 415]. The major challenge faced is to preserve the stable physicochemical properties of the desired nanomedicine, such as drug loading efficiency, size, and zeta potential for each batch [415, 416]. Even the smallest of changes in the mentioned properties could massively affect the therapeutic efficacy, biocompatibility, and biodistribution of the nanomedicine [405].

Manufacturing nanomedicines consistently and at scale remains a major challenge due to their complex structures and sensitivity to production conditions. For example, a research Lipsa et al. [417] studied two commercially available nanosimilar formulations of Doxil, a liposomal based nanomaterial encapsulating doxorubicin for cancer therapy. The formulations, named Dox1 and Dox2, were investigated together with their empty carriers (Dox1C and Dox2C) and free DOX.

While Dox1 and Dox1C exhibited stable and comparable size with low polydispersity similar to that of a standard Doxil (around 85 nm), Dox2 and Dox2C exhibited greater, highly varied particle sizes with higher polydispersity. Sophisticated characterization techniques like asymmetric flow field-flow fractionation (AF4) and analytical ultracentrifugation results revealed that Dox2 consisted of 13% DOX outside liposomal composition, while Dox1 had negligible amounts of DOX outside its liposomal composition. Additionally, Dox2 samples exhibited endotoxin contamination and induced a much stronger toxicity and immune responses in cell-based assays compared to Dox1. These differences between nanosimilar medicines underscore the need for stringent quality control policies, reliable manufacturing protocols, and rigorous regulatory oversight to ensure safety and consistency in nanomedicine production. Without standardized processes, batch variability can severely impact therapeutic outcomes and patient safety.

Apart from that, sophisticated manufacturing technologies involving microfluidic devices and continuous reactors are being investigated to expand advancements in terms of scalability and reproducibility[418, 419]. The mentioned technologies provide much better governance over the chemical reaction and uniform reproducibility of nanomedicines. Progress in the advancement of analytical techniques and methods has been quite slow, thereby posing a challenge in nanomedicine characterization [405]. Conventional analytical tools prove to be inadequate to fully determine the physicochemical properties of nanostructures [420]. This has led to the birth of a unique characterization technique, AF4 integrated with multi-angle light scattering (MALS), which can assess multiple properties of NPs such as drug loading, stability, and size of the synthesized nano-formulations [421–423].

### Biological hindrances and immunogenicity

In nanomedicine-based cancer therapy, one of the major hurdles is overcoming biological hindrances to effectively target the tumor and enhance the nanomaterial’s penetration. Over the years EPR effect has been considered a key targeting strategy for the delivery of NPs for cancer therapy, but the same has fallen under scrutiny as researchers have reported EPR effect poses heterogeneous accumulation for different types of cancer [424]. This variance in accumulation arises due to the stromal density, poor vasculature, and interstitial fluid pressure that hindering deep tumor penetration [71]. The other major problem is off-targeting due to lack of targeting ligands [425]. To overcome this issue, researchers have been actively employing and testing various active targeting techniques to enhance tumor targeting and penetration. This involves decorating the NPs with targeting ligands for effective conjugation with specific receptors on cancer cells. The most popularly used targeting materials are FA, peptides, and antibodies, which not only help NPs to target cancer but also enhance tumor penetration [426–431]. Additionally, stimuli responsive nanoparticles have been extensively scrutinized which responds to tumor associated cues such as low pH, redox gradient, and certain enzymes [432]. Biological components/cell-mediated delivery of therapeutic cargo has shown promising results in tumor targeting and accumulation [433].

Apart from biological barriers affecting targeting efficiency of nanomedicine, the other biological barrier posed on nanomedicine is by the immune system, and their interaction may prove to be a double-edged sword in cancer immunotherapy. Though a minor activation of the immune system has proven to suppress tumors, overactivation may lead to excessive inflammatory response and rapid clearance via opsonisation and phagocytic cells. This is popularly known as accelerated blood clearance (ABC) and can significantly affect circulation time, as a result, drastically reducing the therapeutic efficiency of nanomedicine [434, 435]. Studies have shown surface modification of NPs with PEG via PEGylation to improve circulation time by reducing protein adsorption by NPs, but in contradiction, some research states a possible activation of the complement system via the lectin pathway [435, 436]. Thus focus has shifted to the development and coating of zwitterionic polymers, which have shown enhanced circulation with reduced complement activation [437, 438].

## Conclusions

Despite advancements in the recent past in therapeutics, cancer remains a global threat, with an increase in incidence and high mortality rates. This is primarily attributed to the systemic toxicity posed by therapeutic molecules, drug resistance, and inadequate knowledge of the specificity of the tumor. The complexities associated with organ-specific TME in lung cancer, GBM, and HCC pose a major challenge for the employment of traditional immunotherapy. However, nanomedicines have paved the path for innovative therapy of cancer by leveraging distinctive and irreplaceable physicochemical properties to deliver drugs, facilitate combination therapy, and regulate immune response [439]. The use of nanoarchitectures in therapy has proven promising by aiding chemotherapy, magnetic field-induced dynamic and thermal therapy, photothermal and dynamic therapy, and immunotherapy to circumvent drug resistance and improve cancer prognosis. Organ-targeted nanoplatforms not only deliver therapeutic agents and immune adjuvants to immunosuppressive TME but also regulate spatiotemporal activation of the immune system. This involves transforming cold tumors into hot ones and lesions that are recognizable by immune cells, leading to improved clinical outcomes [440]. Moving forward, to resolve the issue of organotropism, designing smart nanomedicines with advanced and unique capabilities that can detect and exploit local TME conditions, such as hypoxia, pH, and enzymes, would not only be instrumental in winning tumor suppression but also hinder tumors immunity from becoming robust [441]. The rapid evolution and convergence of science and technologies such as sequencing, single-cell profiling, and artificial intelligence would accelerate the pace for tailored nanovaccines for each individual and tumor type [442].

Nonetheless, NPs have their cons, which include manufacturing and scalability, biological hindrances, complexities associated with their regulatory standards, and immunogenicity. However, in the next generation, primary focus must be laid on the development of nanomedicine with unique designs and advanced manufacturing techniques to overcome the current challenges and accelerate real-time clinical use of nanomedicines for cancer immunotherapy. As nanotechnology grows, organ-specific immunotherapeutic strategies are not only on the verge of revolutionizing oncology but also paving the way for tumor therapy which till date is deemed intractable.
